# Inoculation fermentation improves the nutritional quality and flavor profile of Chinese traditional fermented okara (Meitauza): a comparison with a commercial benchmark

**DOI:** 10.3389/fmicb.2026.1846638

**Published:** 2026-06-08

**Authors:** Muhammad Faisal, Zetian Cai, Guihong Zhao, Jian Liu, Fengzhen Chen, Bo Wang, Chunxia Pang, Yuxing Guo, Haibo Luo, Huibo Song

**Affiliations:** 1 College of Agricultural and Biological Engineering (College of Tree Peony), Heze University, Heze, China; 2 School of Food Science and Pharmaceutical Engineering, Nanjing Normal University, Nanjing, China

**Keywords:** antioxidant, aromas, microbial, quality, soybean residue

## Abstract

**Introduction:**

This study compared the effects of inoculated fermentation with pure *Mucor racemosus* (MMtz), *Bacillus subtilis* (BMtz), or *Actinomucor elegans* (AMtz) strains or a mixed culture of *M. racemosus* and *B. subtilis* (MBMtz) on the transformation of soybean residue (okara) into Meitauza relative to a commercially sourced traditional Meitauza (TMtz) used as an industry benchmark.

**Methods:**

Inoculated fermentation markedly improved nutritional quality. The BMtz sampled exhibited the highest soluble protein (31.25 g/kg) and free amino acid contents (141.00 g/kg). The MBMtz samples exhibited synergistically increased reducing sugar levels (38.20 g/kg) and the highest antioxidant capacity with a 57% hydroxyl radical scavenging rate and stable iron-reducing power. Gas chromatography–mass spectrometry analysis identified 242 volatile compounds that contributed to differential flavors.

**Results:**

The MBMtz samples exhibited the most complex and desirable aroma, which was characterized by fruity esters (12.77%) and fresh alcohols (14.86%). The pyrazines were abundant in BMtz samples (23.00%), conferring roasted and nutty aromas. *A. elegans* maintained a slightly alkaline environment and contributed unique volatiles, while *M. racemosus* was an effective single inoculum.

**Discussion:**

These findings indicate that targeted inoculation is a useful strategy to standardize and optimize Meitauza production, promoting soybean processing waste into a nutritionally enhanced, flavor-rich, and functional food product.

## Graphical abstract

**Figure:**
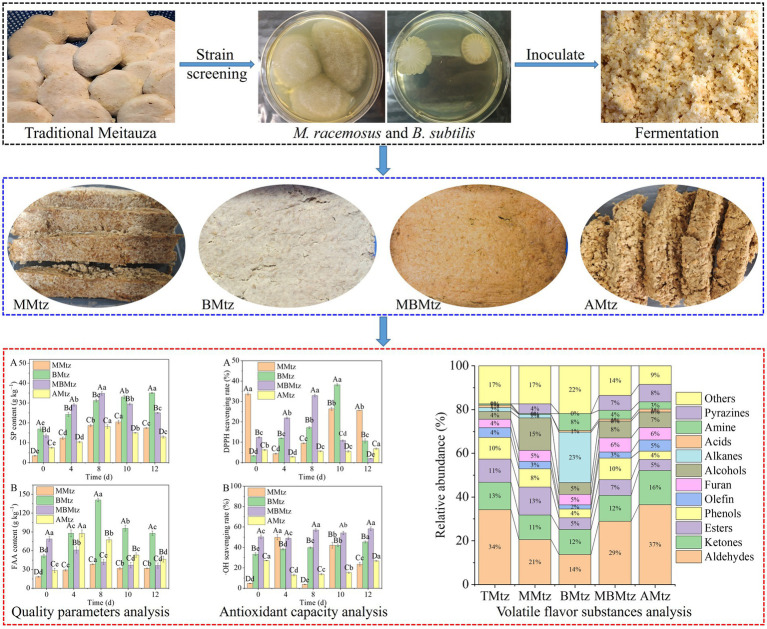

## 1 Introduction

Soybean (*Glycine max* L. Merr.) processing generates vast quantities of by-products, especially fresh soybean residue (okara). Approximately 1.1–1.2 kg of okara is produced for each kilogram of soybeans processed into soy milk or tofu (Usman et al., 2025). In China, the consumption of soy-based foods, such as soy milk, soybean flour, and plant-based meat analogs has been increasing. The annual okara production is more than 10 million tons in China. Okara is highly perishable owing to its high moisture content (80–85%), undergoing spoilage within 6–8 h at ambient temperature (Yu et al., 2024). Most okara is sold cheaply for animal feed, composted, or discarded, contributing to resource wastage and environmental pollution.

However, okara is a nutritionally rich resource, containing 18–23% crude protein, 11–15% fat, and up to 55% dietary fiber on a dry matter basis (Samarasiri and Chen, 2025). Additionally, okara is a source of essential amino acids, isoflavones, saponins, minerals, and vitamins (Canaan et al., 2022). These bioactive components exert beneficial physiological effects, including cholesterol-reducing, anti-hyperglycemic, and gut microbiota-modulating effects. Hence, okara is a promising substrate for value-added food development (Yu et al., 2024).

The traditional Chinese fermented food Meitauza utilizes microbial fermentation to transform low-value substrates into nutrient-dense, bioactive products. During fermentation, microbial enzymes hydrolyze proteins, fibers, and lipids in soybean residue into small, bioavailable molecules, while secondary metabolites contribute to desirable aromas by masking raw “beany” aromas (Zhang et al., 2024). Thus, fermented okara improves palatability and acquires biofunctional properties, such as antioxidant, anti-diabetic, and hypocholesterolemic activities. However, previous studies on spontaneous Meitauza fermentation have focused on microbial taxonomy or gross nutritional evaluation (Tuly and Ma, 2024). Limited studies have examined the ability of starter strains to drive nutrient conversion, flavor formation, and functional enhancement.

Recent studies have characterized the microbial diversity of spontaneous Meitauza fermentation using culture-dependent and sequencing approaches (Shen et al., 2024; Li et al., 2024). This complex microbial community comprises lactic acid bacteria, yeasts, and *Bacillus* spp. These microbes are directly involved in nutrient metabolism and the biosynthesis of flavor compounds (Tkaczewska et al., 2022). Previous studies have focused on microbial taxonomy or overall nutritional properties. However, the effect of specific microbial strains on nutrient metabolism, the correlation between microbial metabolism and flavor development, and the potential to design inoculated fermentations for controlled product quality have not been examined. Furthermore, limited studies have systematically screened the functional strains from soybean residue fermentation, restricting the scope of microbial resource utilization for process optimization.

This study aimed to elucidate the effects of inoculated fermentation with selected microbial strains on the nutritional composition and flavor characteristics of soybean residue-based Meitauza. The mechanistic correlation between microbial metabolism, nutrient transformation, and flavor profile was elucidated using integrated microbial, biochemical, and metabolomic analyses. The findings of this study offer a scientific basis to improve the value of soybean processing by-products through controlled fermentation, contributing to sustainable food innovation and standardized production of functional traditional foods.

## 2 Materials and methods

### 2.1 Materials and treatment

#### 2.1.1 Materials

Fresh soybean residue and ‌*Actinomucor elegans*‌ were provided by Yangzhou Sanhe Simei Pickled Vegetables Co., Ltd. (Yangzhou, Jiangsu, China), and stored at −40 °C for further use. The resulting okara comprised 224 g/kg protein, 126 g/kg lipid, and 553 g/kg dietary fiber (dry basis) (Table 1). Traditional Meitauza (TMtz) was purchased from Hubei Xianning Wangye Agricultural Products Co., Ltd. (Chongyang, Xianning, Hubei, China) and used as a commercial industry benchmark for comparison. Note: TMtz differed from the inoculated samples in raw material origin, sterilization status, moisture content, fermentation vessel, temperature, and duration; it should be interpreted as a commercial reference rather than a matched natural fermentation control. A laboratory-prepared spontaneous fermentation control using non-sterilized okara from the same batch is recommended in future studies.

**Table 1:** Quality indices and antioxidant capacity of fresh soybean residue and traditional Meitauza.

Indices	Fresh soybean residue	Traditional Meitauza
Water content/%	84.43 ± 0.62	5.12 ± 0.04
pH	6.80 ± 0.05	6.66 ± 0.03
Soluble protein (g kg^−1^)	4.91 ± 0.88	4.59 ± 0.38
Free amino acids (g kg^−1^)	2.86 ± 0.38	27.44 ± 1.68
Crude fat content (g kg^−1^)	2.50 ± 0.40	1.46 ± 0.01
Reducing sugar content (g kg^−1^)	6.44 ± 0.93	38.81 ± 1.83
Titratable acids (g kg^−1^)	1.20 ± 0.17	5.40 ± 0.29
DPPH scavenging rate (%)	22.37 ± 3.79	50.20 ± 4.07
Hydroxyl radical scavenging rate (%)	57.37 ± 4.19	51.39 ± 3.94
Fe^2+^ scavenging rate (%)	6.21 ± 0.35	6.21 ± 1.81

Data are expressed as mean ± SD of triplicate assays.

#### 2.1.2 Preparation of microbial suspension

*Bacillus subtilis*, *Mucor racemosus* Fresenius, and *A. elegans* strains previously isolated from TMtz (Pang et al., 2023) were used as pure starters. All strains were cultured in sterile wheat-bran medium under the following conditions: *B. subtilis*: 37 ± 1 °C for 2 d; *M. racemosus*: 15 ± 1 °C for 5 d; *A. elegans*: 28 ± 1 °C for 3 d. The bacterial cells or fungal spores were washed with 8.5 g/L sodium chloride and filtered through sterile gauze. The density of bacterial cells and fungal spores was adjusted to 1 × 10^8^ CFU/mL (*B. subtilis*) and 1 × 10^6^ spores/mL (for both molds), respectively.

#### 2.1.3 Fermentation of Meitauza

The water contents of soybean residue were adjusted to 846.5 g/kg, 763.4 g/kg, and 677.1 g/kg for inoculating *B. subtilis*, *M. racemosus*, and *A. elegans*‌, respectively. The soybean residues (500 g) were placed in a 1 L conical flask and sterilized at 121 °C for 15 min. Next, the samples were inoculated with 18.8 mL/kg *B. subtilis* (1.0 × 10^8^ CFU/mL), 22.8 mL/kg *M. racemosus* (1.0 × 10^6^ spores/mL), or 43.1 mL/kg *A. elegans*‌ (1.0 × 10^6^ spores/mL), sealed, and fermented. The fermentation temperatures for *B. subtilis*, *M. racemosus*, and *A. elegans*‌ were 37 ± 1 °C, 15 ± 1 °C, and 28 ± 1 °C, while the fermentation durations were 60, 137, and 74 h, respectively. For mixed fermentation, *M. racemosus* suspension was inoculated into sterilized soybean residue and incubated at 15 °C for 137 h. Next, the water content was adjusted to 846.5 g/kg, followed by inoculation with *B. subtilis* suspension. Fermentation was performed at 37 °C for 60 h. After the pre-fermentation process, the soybean residue was compacted and subjected to post-fermentation incubation at 20 ± 1 °C to yield *B. subtilis-*fermented Meitauza (BMtz), *M. racemosus-*fermented Meitauza (MMtz), and *A. elegans*-fermented Meitauza (AMtz), and *B. subtilis* + *M. racemosus-*fermented Meitauza (MBMtz) samples. Three biological replicates were established in each group. The optimal fermentation conditions used were based on the results of preliminary experiments (Supplementary Tables S1–S9; Supplementary Figures S1–S3).

### 2.2 Sensory analysis

Sensory evaluation was performed following the methods of Lim, (2011) with minor modifications. A trained panel of 10 assessors (five males and five females; aged 21–45 years) evaluated key sensory attributes, including appearance, color, odor, and texture. Before assessment, the panelists were familiarized with the characteristic sensory profile of Meitauza and standardized evaluation protocols. The overall acceptability was rated on a 9-point hedonic scale (1 = dislike extremely, 9 = like extremely). All panelists provided written informed consent. The optimal post-fermentation time point for each group was defined as the day on which overall acceptability score and SP content jointly reached their highest values. Quantitative scores (mean+/-SD, n = 30) are reported in revised Supplementary Table S10. Three Meitauza samples per treatment were placed on individual white enamel plates for visual assessment in individual sensory booths under ambient lighting at room temperature. The panelists evaluated four samples at each time.

### 2.3 Soluble protein (SP) and free amino acid (FAA) evaluation

The Meitauza sample (2 g) was ground with 20 mL of deionized water and extracted at 30 °C for 20 min. The homogenate was centrifuged at 4,000 *× g* and 4 °C for 20 min. The supernatant was used for the analysis.

The SP content was determined using the Bradford method [Note: The Bradford assay detects proteins/peptides > = ~3–5 kDa only; free amino acids and short peptides are invisible to this assay. The ninhydrin assay measures all primary alpha-amino groups as leucine equivalents. The two assays measure fundamentally different analyte pools and their g/kg values cannot be compared on a mass basis (Gao et al., 2024) with Coomassie Brilliant Blue G-250. The supernatant (0.1 mL) was incubated with 0.9 mL of deionized water and 5 mL of 0.1 g/L of Coomassie Brilliant Blue G-250 (prepared in 50 mL of 95% ethanol and 100 mL of 85% orthophosphate) at 30 °C for 5 min. The absorbance of the reaction mixture at 595 nm was measured using a UV-2800A spectrophotometer (Unico (Shanghai) Instruments, Shanghai, China). The SP content was calculated based on the absorbance values and a bovine serum albumin standard curve (*y* = 0.0065x + 0.0381, *R*^2^ = 0.9885). The results are expressed as grams per kilogram (g/kg) of Meitauza.

The FAA content was determined using the ninhydrin colorimetric methods (Kong et al., 2025a) with minor modifications. The supernatant (0.1 mL) was incubated with a 2.9 mL of deionized water, 1 mL of 20 g/L of ninhydrin (containing 500 mL/L of 95% ethanol, 500 mL of 67 mmol/L phosphate buffer (pH 8.04), and 0.8 g/L stannous chloride), and 1 mL of phosphate buffer in boiling water for 15 min and cooled to room temperature. The absorbance of the sample at 568 nm was measured. The FAA content was calculated using a leucine standard curve (*y* = 0.0015x + 0.0005, *R*^2^ = 0.9983). The results are expressed as g/kg.

### 2.4 Crude fat and reducing sugar (RS) evaluation

The crude fat content was determined following the methods of Sui et al. (2025) with minor modifications. After drying at 60 °C, the Meitauza sample (2 g) was extracted with petroleum ether (boiling point: 50 °C–60 °C) for 4 h using an automatic fat extraction instrument (SOX406, Shandong Haineng Scientific Instrument Co., Ltd., Dezhou, Shandong, China). The fat content of the mold bean residue products was calculated according to the fat yield as follows:


$$\mathrm{Cf}\;(g/\mathrm{kg})=\frac{m_{1}-m_{0}}{m}\times 100$$ (1)

where *Cf* is crude fat content, m_1_ is the weight of crude fat in receiving bottles and samples (g); m_0_ is the weight of the receiving bottle (g); m is the weight of the sample (g).

The RS content was measured using a modified dinitrosalicylic acid (DNS) method, following the protocols of Miller (1959). Briefly, the sample (2 g) was homogenized in 20 mL of deionized water and centrifuged at 8,000 *g* for 20 min. Next, the supernatant (0.1 mL) was incubated with 1.9 mL of deionized water and 2 mL of 6.5 g/L DNS reagent (containing 1 mol/L NaOH and 45 g/L glycerol) and boiled for 5 min. After cooling, the solution was diluted to 25 mL with deionized water. The absorbance of the sample at 540 nm was measured. The RS content was calculated from the glucose standard curve (*y* = 3.454x − 0.0384, *R*^2^ = 0.9995). The results are expressed as g/kg.

### 2.5 pH and titratable acidity (TA) evaluation

The Meitauza sample (2 g) was ground with 20 mL of deionized water and incubated at 30 °C for 20 min. The sample was centrifuged at 8,000 *g* for 20 min. The pH of the supernatant was measured using an FE28 pH meter (Mettler Toledo Instruments (Shanghai) Co., Ltd., Shanghai, China).

The TA was determined following the modified methods of Kong et al. (2025b). Briefly, the Meitauza sample (1 g) was homogenized with 40 mL of 80% ethanol solution and subjected to ultrasound-assisted extraction for 30 min. The homogenate was centrifuged at 10,000 *g* and 4 °C for 20 min. The supernatant (20 mL) was mixed with two drops of phenolphthalein indicator. The sample was titrated with a 50 mmol/L sodium hydroxide standard solution until a slight red color formed and persisted for 30 s. Lactic acid is the predominant organic acid in Meitauza. The results are expressed as grams of lactic acid equivalents per kilogram of Meitauza (g/kg).

### 2.6 Antioxidant activity evaluation

The Meitauza sample (2 g) was ground with 40 mL of 80% ethanol solution and extracted at 50 °C for 4 h. The homogenate was centrifuged at 8,000 *g* and 4 °C for 20 min. The supernatant was used for analysis.

The ability of Meitauza to scavenge 2,2-diphenyl-1-picrylhydrazyl (DPPH) and hydroxyl radicals was assessed following the methods of Kong et al. (2025b) with slight modifications. To assess the DPPH radical scavenging capacity, the supernatant (1 mL) was incubated with 1 mL of freshly prepared anhydrous ethanol solution of DPPH (0.2 mmol/L) for 30 min in the dark. In the blank samples, the supernatant was replaced with an equal volume of deionized water. The absorption of the sample at 517 nm (A_517_) was measured. The DPPH scavenging rate was calculated as follows:


$$\begin{array}{l} \text{DPPH scavening rate}\;(\%)\\ =\frac{A517\;(\text{blank})-A517\;(\text{sample})}{A517\;(\text{blank})}\times 100\% \end{array}$$ (2)

To evaluate the hydroxyl radical scavenging ability, the supernatant (1 mL) was incubated with 1 mL of 2.5 mol/L phenanthroline solution, 1 mL of 200 mmol/L phosphate buffer (pH 6.6), 1 mL of 7.5 mmol/L FeSO_4_, and 1 mL of 10 g/L hydrogen peroxide (H_2_O_2_) at 37 °C for 1.5 h. The absorbance of the sample at 536 nm (A_536_) was measured. In the blank sample, the supernatant was replaced with an equal volume of deionized water. Meanwhile, H_2_O_2_ was replaced with an equal volume of deionized water in the control samples. The hydroxyl radical scavenging rate was calculated as follows:


$$\begin{array}{l} \text{Hydroxyl radical svagening rate}\;(\%)\\ =\frac{A536\;(\text{sample})–A536\;(\text{blank})}{A536\;(\text{control})–A536\;(\text{blank})}\times 100\% \end{array}$$ (3)

The iron-reducing activity was determined using the potassium ferricyanide methods (Kong et al., 2025b; Wang et al., 2022). Briefly, the supernatant (0.5 mL) was incubated with 0.5 mL of phosphate buffer and 0.5 mL of 10 g/L potassium ferricyanide at 50 °C for 20 min and rapidly cooled. The mixture was then incubated with 0.5 mL of 100 g/L trichloroacetic acid solution for 5 min. Next, the reaction mixture (1 mL) was incubated with 1 mL of deionized water and 1 mL of 1 g/L ferric chloride for 10 min. The absorbance of the sample at 700 nm (A_700_) was measured. In the blank control, the supernatant was replaced with an equal volume of deionized water. The iron-reducing activity was calculated as follows:


$$\begin{array}{l} \text{Iron}-\text{reducing activity}\;(\%)\\ =\frac{A700\;(\text{sample})–A700\;(\text{blank})}{A700\;(\text{blank})}\times 100\% \end{array}$$ (4)

### 2.7 Flavor component analysis

Volatile components were analyzed using headspace solid-phase microextraction coupled with gas chromatography–mass spectrometry (GC–MS), following the methods of Lancioni et al., (2022) with minor modifications. The sample (approximately 3 g) was placed in a 20 mL headspace vial with 3 mL saturated NaCl solution, equilibrated at 80 °C for 30 min, and extracted using a 50/30 μm DVB/CAR/PDMS fiber for 30 min. Desorption was performed in splitless mode for 5 min at 250 °C. GC–MS analysis was performed under the following conditions: column: HP-5MS (30 m × 0.25 mm × 0.25 μm); carrier gas: helium; carrier gas flow rate: 1.0 mL/min; oven temperature program: from 45 °C (4 min) to 130 °C (6 °C/min) and 240 °C (10 °C/min, 8 min). Compounds were identified by matching mass spectra against the NIST17 library, supplemented by linear retention indices (RIs) calculated from a co-injected n-alkane series (C6-C30) and compared against the NIST WebBook and Adams RI database (threshold: ± 20 RI units); compounds exceeding this threshold were reclassified as “tentatively identified.” For semi-quantification, 2-methyl-3-heptanone was used as an internal standard (1 ug/mL). Estimated concentrations (ug/kg) and odor activity values (OAVs) for the 30 most abundant compounds are in Supplementary Table S12. Relative peak area percentages are retained in Table 2 for cross-group comparison. For 12 key aroma-active compounds (including 2,5-dimethylpyrazine, 2,3,5-trimethylpyrazine, benzaldehyde, nonanal, 1-octen-3-ol, 2-pentyl furan, and 2,4-di-tert-butylphenol), authentic reference standards (Sigma-Aldrich, > = 98% purity) were co-injected under identical GC–MS conditions to confirm identification by both mass spectral match and RI alignment. Confirmed compounds are marked in the revised Table 2.

**Table 2:** Volatile flavor compounds of different Meitauza after fermentation.

No.	Volatile flavor compounds	Samples					Retention time	Chemical abstracts service	Molecular formula
		TMtz	MMtz	BMtz	MBMtz	AMtz			
Esters									
1	Benzoic acid, 4-[1-oxo-2-(1-pyrrolidinyl)ethyl]amino-, methyl ester	1.35	—	—	—	—	10.90	296,245–96-0	C14H18N2O3
2	3-Butenoic acid, ethyl ester	0.81	—	—	—	—	5.85	1,617-18-1	C6H10O2
3	Methyl formate	0.69	—	—	—	—	8.91	107–31-3	C2H4O2
4	2-Furanmethanol, tetrahydro-, acetate	0.60	—	—	—	—	17.58	637–64-9	C7H12O3
5	Methyl 2-butynoate	0.57	—	—	—	—	4.40	23,326–27-4	C5H6O2
6	2,2,4-Trimethyl-1,3-pentanediol diisobutyrate	0.54	2.22	—	5.31	2.30	25.23	6,846-50-0	C16H30O4
7	Vinyl crotonate	0.45	—	—	—	—	10.88	14,861–06-4	C6H8O2
8	Propanoate, (Z)-3-hexen-1-ol	—	1.19	—	—	—	4.43	33,467–74-2	C9H16O2
9	Dodecanoic acid, 3-hydroxy-, ethyl ester	—	1.02	—	—	—	17.72	126,679–28-5	C14H28O3
10	Butanethioic acid, S-methyl ester	—	0.96	—	—	—	6.32	2,432-51-1	C5H10OS
11	(2E,6E,10E)-3,7,11,15-Tetramethylhexadeca-2,6, 10,14-tetraen-1-yl formate	—	0.73	—	—	—	23.49	125,456–63–5	C21H34O2
12	Phosphorous acid, dibutyl ester	—	0.61	—	—	—	13.84	109–47-7	C8H19O3P
13	1,5-Dimethyl-1-vinyl-4-hexenyl butyrate	—	0.37	—	—	—	12.14	78–36-4	C14H24O2
14	Dihydro-5-pentyl-2(3H)-Furanone	—	—	1.62	—	2.04	19.46	104–61-0	C9H16O2
15	2-Pentenoic acid, ethyl ester	—	—	0.78	—	—	12.01	2,445-93-4	C7H12O2
16	Benzeneacetic acid, ethyl ester	—	—	0.70	—	—	16.27	101–97-3	C10H12O2
17	Ethyl trans-2-pentenoate	—	—	0.65	—	—	22.15	24,410–84-2	C7H12O2
18	Benzeneacetic acid, methyl ester	—	—	0.56	—	—	14.39	101–41-7	C9H10O2
19	cis-3-Hexenyllactate	—	—	0.48	—	—	4.60	61,931–81-5	C9H16O3
20	Propanoic acid, 2-benzyl-2-hydroxy-3-phenyl-, 2-(1-piperidyl)ethyl ester	—	—	0.48	—	—	10.91	97,508–25-3	C23H29NO3
21	Propanoic acid, ethenyl ester	—	—	—	5.72	—	8.71	105–38-4	C5H8O2
22	Propanoic acid, 2-oxo-, methyl ester	—	—	—	0.66	—	8.82	600–22-6	C4H6O3
23	2-Butenedioic acid (Z)-, monoethyl ester	—	—	—	0.58	—	12.01	3,990-03-2	C6H8O4
24	Tetradecanoic acid, ethyl ester	—	—	—	0.33	—	29.66	124–06-1	C16H32O2
25	Hexadecanoic acid, ethyl ester	—	—	—	0.18	—	35.56	628–97-7	C18H36O2
26	n-Caproic acid vinyl ester	—	—	—	—	2.91	8.77	3,050-69-9	C8H14O2
27	Ethanethioic acid, S-(2-furanylmethyl) ester	—	—	—	—	1.29	9.00	13,678–68-7	C7H8O2S
28	Hydrogen isocyanate	—	—	—	—	0.56	9.99	75–13-8	CHNO
29	4-Methoxycarbonyl-4-butanolide	—	—	—	—	0.50	10.79	3,885-29-8	C6H8O4
30	Acetic acid, isothiocyanato-, ethyl ester	—	—	—	—	0.47	23.27	24,066–82-8	C5H7NO2S
31	Trimethylene oxide	—	—	—	—	0.46	9.98	503–30-0	C3H6O
Total		5.02	7.10	5.27	12.77	10.53			
Alcohols									
32	1-Nonen-3-ol	2.44	—	—	—	—	8.65	21,964–44-3	C9H18O
33	1-Dodecanol	1.96	—	—	—	—	11.27	112–53-8	C12H26O
34	(S)-(+)-5-Methyl-1-heptanol	1.14	—	—	—	—	8.03	57,803–73-3	C8H18O
35	2-Naphthalenemethanol, decahydro-alpha, alpha, 4a-trimethyl-8-methylene-, [2R-(2.alpha., 4a.alpha., 8a.*β*.)]-	0.67	—	—	—	—	26.92	473–15-4	C15H26O
36	4,8-Dimethyl-1,7-Nonadien-4-ol	0.46	—	—	—	—	17.20	17,920–92-2	C11H20O
37	[1aR-(1a.alpha.,4.β.,4a.β.,7.alpha.,7a.*β*.,7b.alpha.)]-, decahydro-1,1,4,7-tetramethyl-, 4aH-Cycloprop[e]azulen-4a-ol	0.35	—	—	—	—	24.66	5,986-49-2	C15H26O
38	(E, Z)-3,6-Nonadien-1-ol	—	4.00	—	—	—	10.10	56,805–23-3	C9H16O
39	Cyclobutanol	—	1.25	—	—	—	4.38	2,919–23–5	C4H8O
40	1-Nonen-4-ol	—	1.22	—	0.96	1.44	12.00	35,192–73–5	C9H18O
41	1-Hexanol	—	1.18	—	—	—	5.91	111–27-3	C6H14O
42	2-Ethyl-cyclobutanol	—	—	1.67	—	—	4.37	35,301–43-0	C6H12O
43	2-Nonanol	—	—	1.30	—	—	12.16	628–99-9	C9H20O
44	1-Hexen-3-ol	—	—	0.93	—	—	8.71	4,798-44-1	C6H12O
45	(1,4-Dihydrophenyl)-methanol	—	—	0.77	—	—	18.26	25,372–69-4	C7H10O
46	3-Ethyl-2-pentanol	—	—	0.68	—	—	6.59	609–27-8	C7H16O
47	1-Octen-3-ol	—	—	—	10.93	—	8.71	3,391-86-4	C8H16O
48	1-Methyl-2-(1-methylethenyl)-, cis-cyclobutaneethanol	—	—	—	1.17	—	22.36	30,820–22-5	C10H18O
49	2-Methyl-1,5-heptadiene-3,4-diol	—	—	—	0.55	—	8.85	22,726–05-2	C8H14O2
50	N-Methyl-L-prolinol	—	—	—	0.47	—	9.32	34,381–71-0	C6H13NO
51	Propargyl alcohol	—	—	—	0.42	—	12.25	107–19-7	C3H4O
52	1-(2-Furanyl)-3-butene-1,2-diol	—	—	—	0.37	—	16.99	19,261–13-3	C8H10O3
53	Cyclohexanethiol, 2,5-dimethyl-, acetate	—	—	—	—	1.06	17.14	4,186-80-5	C10H18OS
54	1-butyl-vyclohexanol,	—	—	—	—	0.64	19.64	5,445-30-7	C10H20O
55	2-phenoxy-1-Propanol	—	—	—	—	0.45	14.11	4,169-04-4	C9H12O2
Total		7.01	7.66	5.35	14.86	3.59			
Ketones									
56	2-Undecanone	2.91	—	0.46	—	—	17.59	112–12-9	C11H22O
57	2-Nonanone	2.08	—	1.08	—	—	11.90	821–55-6	C9H18O
58	2-butyl-cyclohexanone	1.77	1.92	1.62	—	—	16.83	1,126-18-7	C10H18O
59	3-Octen-2-one	1.10	2.83	—	0.94	1.06	10.35	1,669-44-9	C8H14O
60	3,5-Dimethyldihydropyran-2,6-dione	1.06	—	—	—	—	4.56	7,446-84-6	C7H10O3
61	2-Hexanone	0.92	—	—	—	—	11.89	591–78-6	C6H12O
62	1-Cyclopropyl-2-propen-1-one	0.72	—	—	—	—	13.84	59,819–62-4	C6H8O
63	4-(2,6,6-Trimethyl-1-cyclohexen-1-yl)-3-buten-2-one	0.71	—	—	—	—	22.60	14,901–07-6	C13H20O
64	trans-.β.-Ionone	0.68	—	—	—	—	22.60	79–77-6	C13H20O
65	Methyl vinyl ketone	0.64	—	—	—	—	8.62	78–94-4	C4H6O
66	4-Heptanone	0.55	—	—	—	—	6.43	123–19-3	C7H14O
67	4,5,6,7-Tetrahydro-2-indanone	0.55	—	—	—	—	10.90	20,990–33-4	C9H12O
68	3-Nonen-2-one	0.51	0.79	—	0.96	1.12	13.26	14,309–57-0	C9H16O
69	3-Acetyl-2,6-heptanedione,	0.49	—	—	—	—	12.39	29,214–57-1	C9H14O3
70	Isophorone	0.48	—	—	—	—	9.02	78–59-1	C9H14O
71	2(5H)-Furanone	0.43	—	—	—	—	10.89	497–23-4	C4H4O2
72	[1S-(1.alpha.,4.alpha.,5.alpha.,11a.alpha.)]-, decahydro-4-(2-propenyl)-, 1,5-Methano-8H-pyrido[1,2-a][1,5]diazocin-8-one	—	2.53	—	1.08	—	8.10	550–43-6	C14H22N2O
73	2-Heptanone	—	2.52	—	1.12	—	6.32	110–43-0	C7H14O
74	2-Octanone	—	1.30	—	—	—	6.26	111–13-7	C8H16O
75	1-(2-Aminophenyl)-ethanone	—	—	4.68	—	1.49	17.86	551–93-9	C8H9NO
76	6-Dodecanone	—	—	0.90	—	—	19.65	6,064-27-3	C12H24O
77	5-methyl-3-Hepten-2-one	—	—	0.79	—	—	10.36	5,090-16-4	C8H14O
78	2-Decanone	—	—	0.73	—	1.14	13.91	693–54-9	C10H20O
79	6-Tridecanone	—	—	0.64	—	—	22.20	22,026–12-6	C13H26O
80	2,7-Octanedione	—	—	0.61	—	—	10.23	1,626-09-1	C8H14O2
81	1-(2-Furanyl)-2-butanone	—	—	—	3.29	3.50	8.97	4,208-63-3	C8H10O2
82	4’-Propoxy-2-methylpropiophenone	—	—	—	0.74	—	21.82	64,436–60-8	C13H18O2
83	Pyrolo[3,2-d]pyrimidin-2,4(1H,3H)-dione	—	—	—	0.59	—	7.05	65,996–50-1	C6H5N3O2
84	2-Methoxyphenylacetone	—	—	—	0.57	—	16.73	5,211-62-1	C10H12O2
85	Octahydro-5-hydroxy-8a-methyl-1(2H)-Naphthalenone	—	—	—	0.50	—	19.35	91,965–65-0	C11H18O2
86	1,2-Dihydro-3-(3-methylphenyl)-2-thioxo-quinazolin-4(3H)-one	—	—	—	0.41	—	12.60	37,641–49-9	C15H12N2OS
87	2-n-Hexylcyclopentanone	—	—	—	0.35	—	18.93	13,074–65-2	C11H20O
88	2-n-Heptylcyclopentanone	—	—	—	0.34	—	18.93	137–03-1	C12H22O
89	3(2H)-Furanone, 2-hexyl-5-methyl-	—	—	—	0.28	—	21.43	33,922–66-6	C11H18O2
90	(Z)-Undec-6-en-2-one	—	—	—	—	1.62	17.14	107,853–70-3	C11H20O
91	2-Tetradecanone	—	—	—	—	1.06	17.60	2,345-27-9	C14H28O
92	3-(Hydroxymethyl)-2-nonanone	—	—	—	—	0.98	11.89	67,801–33-6	C10H20O2
93	Acetone	—	—	—	—	0.60	6.40	67–64-1	C3H6O
Total		15.61	11.89	11.51	11.18	12.56			
Phenols									
94	2-(1-Methylpropyl)-phenol	2.01	—	—	0.76	—	18.27	89–72-5	C10H14O
95	2,4-Di-tert-butylphenol	1.77	7.30	1.33	6.33	9.32	23.26	96–76-4	C14H22O
96	4-(1-Methylpropyl)-phenol	—	2.30	2.00	1.30	—	18.26	99–71-8	C10H14O
97	1-Naphthalenol	—	0.35	—	—	—	15.65	90–15-3	C10H8O
98	o-Amino-phenol	—	—	0.68	—	—	6.78	95–55-6	C6H7NO
99	4-Heptyl-phenol	—	—	—	—	0.59	8.10	1987-50-4	C13H20O
Total		3.77	9.95	4.01	8.39	9.91			
Aldehydes									
100	Nonanal	11.83	6.97	1.60	4.33	2.90	12.25	124–19-6	C9H18O
101	(E, E)-2,4-decadienal	4.03	2.84	1.74	0.51	1.88	18.22	25,152–84-5	C10H16O
102	2-Undecenal	3.48	1.87	—	—	—	19.46	2,463-77-6	C11H20O
103	(E)-2-Octenal	3.09	5.65	2.30	2.03	3.01	10.90	2,548-87-0	C8H14O
104	Decanal	2.71	0.56	—	—	—	15.15	112–31-2	C10H20O
105	Octanal	2.63	1.55	—	—	—	9.30	124–13-0	C8H16O
106	(E)-2-Decenal,	2.63	1.70	—	—	—	16.72	3,913-81-3	C10H18O
107	(E)- 2-Nonenal	2.19	1.34	—	1.20	—	13.84	18,829–56-6	C9H16O
108	(E, E)-2,4-Nonadienal	1.20	—	—	—	—	15.39	5,910-87-2	C9H14O
109	(E, Z)-2,4-Decadienal	0.94	0.76	—	—	—	17.60	25,152–83-4	C10H16O
110	2-Butyl-2-octenal	0.72	—	—	—	—	19.73	13,019–16-4	C12H22O
111	2,6,6-trimethyl-1-cyclohexene-1-carboxaldehyde	0.67	—	—	—	—	15.62	432–25-7	C10H16O
112	2,4-dimethyl-2,4-heptadienal	0.42	—	—	—	—	12.84	42,452–48-2	C9H14O
113	Benzaldehyde	—	2.88	5.95	7.35	22.37	8.18	100–52-7	C7H6O
114	(E)-4-Decenal	—	1.43	—	—	—	20.22	65,405–70-1	C10H18O
115	Benzeneacetaldehyde	—	1.37	1.32	0.51	2.49	10.52	122–78-1	C8H8O
116	4-Oxohex-2-enal	—	—	0.82	—	—	8.06	20,697–55-6	C6H8O2
117	Hexanal	—	—	—	3.80	—	4.39	66–25-1	C6H12O
118	1H-Pyrrole-2-carboxaldehyde	—	—	—	0.51	—	10.13	1,003-29-8	C5H5NO
119	5-Heptyldihydro-2(3H)-furanone	—	—	—	0.36	—	19.45	104–67-6	C11H20O2
120	5-Ethylcyclopent-1-enecarboxaldehyde	—	—	—	—	0.92	10.14	36,431–60-4	C8H12O
121	Cyclohexanecarboxaldehyde	—	—	—	—	0.66	13.83	2043-61-0	C7H12O
Total		36.54	28.91	13.73	20.59	34.24			
Acids									
122	Octanoic acid	0.70	—	—	—	—	14.34	124–07-2	C8H16O2
123	Alpha-pyrone-6-carboxylic acid	0.64	—	—	—	—	10.14	672–67-3	C6H4O4
124	.alpha.-Aminoisobutanoic acid	—	0.91	—	—	—	4.48	62–57-7	C4H9NO2
125	2-Methyl-butanoic acid	—	—	0.83	—	—	5.79	116–53-0	C5H10O2
126	5-(1,1-Dimethylethyl)-1,3-Benzenedicarboxylic acid	—	—	—	—	0.59	4.98	2,359-09-3	C12H14O4
127	Benzoylformic acid	—	—	—	—	0.56	11.18	611–73-4	C8H6O3
Total		1.35	0.91	0.83	0.00	1.16			
Furan									
128	2-pentyl-furan	2.66	2.91	5.87	0.36	0.56	8.99	3,777-69-3	C9H14O
129	2-n-Heptylfuran	0.66	—	—	—	—	19.02	3,777-71-7	C11H18O
130	3-Acetyl-2,5-dimethyl furan	—	0.61	—	—	—	12.37	10,599–70-9	C8H10O2
131	3-Phenyl-furan	—	0.55	—	—	—	15.64	13,679–41-9	C10H8O
132	2-Vinylfuran	—	—	1.84	—	—	8.93	1,487-18-9	C6H6O
Total		3.32	4.07	7.71	0.36	0.56			
Pyrazines									
133	Trimethyl-pyrazine	—	—	6.56	1.62	1.76	9.29	14,667–55-1	C7H10N2
134	2,5-Dimethyl-pyrazine	—	—	14.42	—	—	6.82	123–32-0	C6H8N2
135	3-Ethyl-2,5-dimethyl-pyrazine	—	—	1.11	—	—	11.53	13,360–65-1	C8H12N2
136	2,6-Diethyl-pyrazine	—	—	0.91	—	—	11.53	13,067–27-1	C8H12N2
Total		0.00	0.00	23.00	1.62	1.76			
Amines									
137	(2-Aziridinylethyl)amine	2.77	—	0.45	—	—	4.37	4,025-37-0	C4H10N2
138	2-Aminocyanoacetamide	1.85	—	—	—	—	4.57	6,719-21-7	C3H5N3O
139	Dimethyl-cyanamide	0.53	—	—	—	—	11.28	1,467-79-4	C3H6N2
140	Furfurylmethylamphetamine	—	1.69	—	—	—	8.99	13,445–60-8	C15H19NO
141	N-tert-Butylmethylamine	—	1.06	0.89	—	—	8.88	14,610–37-8	C5H13N
142	N-Methyl-1-octanamine	—	—	0.52	—	—	4.37	2,439-54-5	C9H21N
143	N, N-Di(2-propynyl)-N-ethylamine	—	—	—	1.34	—	8.14	13,002–92-1	C8H11N
144	1,2-Bis(4-methoxyphenyl)ethane-1,2-diamine	—	—	—	1.29	—	11.75	51,208–43-6	C16H20N2O2
145	.alpha.-Methyl-1H-imidazole-4-ethanamine	—	—	—	0.71	—	12.25	6,986-90-9	C6H11N3
146	N-(2-methylpropyl)-acetamide	—	—	—	—	3.20	10.00	1,540-94-9	C6H13NO
147	L-Prolinamide	—	—	—	—	0.68	6.53	7,531-52-4	C5H10N2O
148	2-Octanamine	—	—	—	—	0.57	12.24	693–16-3	C8H19N
Total		5.16	2.75	1.86	3.34	4.44			
Alkanes									
149	1-hexyl-3-methyl-Cyclopentane	2.02	—	—	0.56	—	11.27	61,142–68-5	C12H24
150	1,3-Dimethoxy-propane	1.30	—	—	—	—	4.33	17,081–21-9	C5H12O2
151	(S)-2-Butanamine	1.23	—	—	—	—	4.36	513–49-5	C4H11N
152	6-Thiabicyclo[3.1.0]hexane	1.18	—	—	—	—	9.28	285–75-6	C5H8S
153	2,3-Epoxybutane	—	3.34	—	—	—	4.58	3,266-23-7	C4H8O
154	Tetradecane	—	0.81	0.58	—	1.15	20.36	629–59-4	C14H30
155	1-(3-butyloxiranyl)-ethanone	—	0.66	1.08	—	—	14.60	17,257–80-6	C8H14O2
156	2-(Pyrrolidin-1-ylmethyl)pyrrolidine	—	0.58	—	—	—	19.46	195,311–28-5	C9H18N2
157	1,3-Dimethyl-5-(propen-1-yl)adamantane	—	0.54	—	—	—	20.73	57,040–48-9	C15H24
158	7-methyl-pentadecane	—	0.52	—	—	—	27.46	6,165-40-8	C16H34
159	Hexadecane	—	—	1.08	—	—	25.23	544–76-3	C16H34
160	2-Ethyl-1,6-Dioxaspiro[4.4]nonane	—	—	0.93	—	—	11.59	38,401–84-2	C9H16O2
161	Undecane	—	—	0.80	—	—	22.85	1,120-21-4	C11H24
162	2-Methylthiolane, S, S-dioxide	—	—	0.40	—	—	12.25	1,003-46-9	C5H10O2S
163	2-Butyl-1,2-azaborolidine	—	—	—	1.74	—	8.70	5,357-10-8	C7H16BN
164	3-Amino-2,4-dimethylpentane	—	—	—	0.75	—	4.39	4,083–57-2	C7H17N
165	2,6,11-Trimethyl-dodecane,	—	—	—	0.59	—	20.36	31,295–56-4	C15H32
166	2-Methyl-5-propyl-nonane	—	—	—	0.54	—	27.46	31,081–17-1	C13H28
167	1-Ethenyl-1-methyl-2-(1-methylethenyl)-4-(1-methylethylidene)-cyclohexane	—	—	—	0.46	—	23.50	3,242-08-8	C15H24
168	Bicyclo[5.3.0]decane (cis)	—	—	—	0.37	—	27.81	16,189–46-1	C10H18
169	Propylene oxide	—	—	—	—	1.41	9.99	75–56-9	C3H6O
170	(Z)-2,2-Dimethyl-3-(3-methylpenta-2,4-dien-1-yl)oxirane	—	—	—	—	0.67	26.66	33,281–83-3	C10H16O
171	Azetidine	—	—	—	—	0.64	9.00	503–29-7	C3H7N
Total		5.73	6.45	4.87	5.02	3.86			
Olefin									
172	D-Limonene	3.77	—	—	—	—	10.10	5,989-27-5	C10H16
173	5-Hexyl-3,3-dimethyl-cyclopentene	1.12	—	—	—	—	20.41	61,142–66-3	C13H24
174	2,7-Dioxatricyclo[4.4.0.0(3,8)]dec-4-ene	1.01	—	—	—	—	14.84	51,826–18-7	C8H10O2
175	2,2’-Bioxirane	0.65	—	—	—	—	18.94	1,464–53–5	C4H6O2
176	1,2:3,4-Diepoxy-, (±)-butane	0.62	—	—	—	—	18.94	298–18-0	C4H6O2
177	3-Methoxyhex-1-ene	0.43	—	—	—	—	4.46	108,811–41-2	C7H14O
178	Isoledene	0.36	—	—	0.30	—	20.73	95,910–36-4	C15H24
179	3-Phenylthio-1,2,3-triphenylcyclopropene	—	1.76	—	—	—	13.71	137,541–87-8	C27H20S
180	2,6-dimethyl-1-Octene	—	1.51	—	—	—	11.30	6,874-29-9	C10H20
181	5-Methylene-7-Oxabicyclo[2.2.1]hept-2-ene	—	1.21	—	—	—	10.07	105,089–89-2	C7H8O
182	3-Propyl-cyclohexene	—	0.93	—	—	—	19.65	3,983-06-0	C9H16
183	6-Methyl-1-octene	—	0.74	—	—	—	14.19	13,151–10-5	C9H18
184	1-Methoxy-6,6-dimethyl-cyclohex-2-ene	—	0.60	—	—	—	20.22	73,756–33-9	C9H16O
185	Tetrahydro-, (3a.alpha.,3b.alpha.,6a.alpha.,6b.alpha.)- Cyclobuta[1,2-d:3,4-d’]bis[1,3]dioxole	—	—	—	3.58	—	8.71	69,956–59-8	C6H8O4
186	1,2,3-Triphenyl-3-methyl-cyclopropene	—	—	—	0.55	—	9.22	58,310–19-3	C22H18
Total		7.95	6.74	0.00	4.44	0.00			
Others									
187	Propanoic acid, anhydride	1.76	—	0.36	0.47	—	8.66	123–62-6	C6H10O3
188	Pentanoic acid, 2-methyl-, anhydride	1.49	—	—	—	—	8.75	63,169–61-9	C12H22O3
189	2-hexyl-thiophene	1.32	0.52	—	—	—	17.00	18,794–77-9	C10H16S
190	2,3-dihydro-1-methyl-1H-pyrrole	0.88	—	—	—	—	13.84	33,838–11-8	C5H9N
191	Phenylglyoxal	0.82	—	—	—	—	8.11	1,074-12-0	C8H6O2
192	Anisole	0.66	—	—	—	—	18.26	100–66-3	C7H8O
193	Indole	0.59	—	—	0.82	0.81	17.70	120–72-9	C8H7N
194	4,5-dihydro-5,5-dimethyl-1H-pyrazole	0.52	—	—	—	—	10.91	4,320-85-8	C5H10N2
195	Βzole	0.49	—	—	—	—	4.32	105–20-4	C5H9N3
196	2-(4-Methylphenyl)-indolizine	—	2.01	—	—	—	23.26	7,496-81-3	C15H13N
197	Propanoic acid, 2-methyl-, anhydride	—	1.68	—	—	—	8.81	97–72-3	C8H14O3
198	Succinic anhydride	—	1.57	—	—	—	4.39	108–30-5	C4H4O3
199	1a,2,3,3a,4,5,6,7b-Octahydro-1,1,3a,7-tetramethyl-, [1aR-(1a.alpha.,3a.alpha.,7b.alpha.)]-, 1H-cyclopropa[a]naphthalene	—	1.53	—	—	—	20.73	489–29-2	C15H24
200	1,3,2-Dioxathiane, 2,2-dioxide	—	1.19	—	—	—	8.97	1,073-05-8	C3H6O4S
201	4’-Amino-6-methoxyaurone	—	1.10	—	—	—	12.61	77,764–96-6	C16H13NO3
202	1H-Pyrazole-4-carbonitrile	—	0.84	—	—	—	10.10	31,108–57-3	C4H3N3
203	1,4-Diethoxy-benzene	—	0.81	—	—	—	19.01	122–95-2	C10H14O2
204	1,2,4,5-Tetrazine	—	0.74	—	—	0.46	18.21	290–96-0	C2H2N4
205	2-Heptyl-thiophene	—	0.61	—	—	—	16.98	18,794–78-0	C11H18S
206	1,1-Dimethoxy-2-butyne	—	0.52	—	—	—	10.32	22,022–34-0	C6H10O2
207	1,5-Dimethyl-1H-tetrazole	—	0.46	—	—	—	14.19	5,144-11-6	C3H6N4
208	2,5-Dimethylpyrimidine	—	—	13.48	—	—	6.81	22,868–76-4	C6H8N2
209	1,2,4-Triazolo[4,3-b]pyridazine	—	—	2.18	—	—	17.86	274–83-9	C5H4N4
210	5-methyl-1H-tetrazole	—	—	1.41	—	—	5.95	4,076-36-2	C2H4N4
211	2-Methyl-1H-pyrrole	—	—	0.84	—	—	9.32	636–41-9	C5H7N
212	2-Methyl-2H-tetrazole	—	—	0.80	—	—	5.90	16,681–78-0	C2H4N4
213	2,4-Dimethyl-quinazoline	—	—	0.76	0.96	0.85	20.30	703–63-9	C10H10N2
214	5-Acetylpyrimidine	—	—	0.66	—	—	6.79	10,325–70-9	C6H6N2O
215	4-C-methyl-myo-inositol	—	—	0.48	—	—	10.88	472–95-7	C7H14O6
216	S-Benzyl phenylmethanethiosulfonate	—	—	0.47	—	—	18.26	16,601–40-4	C14H14O2S2
217	1-Isobutyl-3-methyl-2-pyrazoline	—	—	0.42	0.38	—	16.99	26,964–53-4	C8H16N2
218	Tetramethyl-pyrazine	—	—	—	2.06	—	11.74	1,124-11-4	C8H12N2
219	Carbonothioic dihydrazide	—	—	—	1.58	—	8.14	2,231-57-4	CH6N4S
220	3’-Amino-6-methoxyaurone	—	—	—	1.58	—	12.60	77,764–95-5	C16H13NO3
221	4-Methyl-quinazoline	—	—	—	1.57	—	18.96	700–46-9	C9H8N2
222	5,6,7,8-Tetrahydro-ouinoline	—	—	—	1.43	—	16.74	10,500–57-9	C9H11N
223	2-Methyl-quinoxaline,	—	—	—	1.24	1.87	18.96	7,251-61-8	C9H8N2
224	4-Methylthiazole	—	—	—	1.04	—	8.85	693–95-8	C4H5NS
225	Azetidine, 1-(1,1-dimethylethyl)-3-methyl-	—	—	—	1.02	—	8.01	55,702–65-3	C8H17N
226	2-Pentyl-piperidine	—	—	—	0.78	—	15.90	33,354–97-1	C10H21N
227	1,2,3,4-Tetrahydro-quinoline	—	—	—	0.58	1.46	16.74	635–46-1	C9H11N
228	1-Methyl-isoquinoline	—	—	—	0.56	—	19.85	1721-93-3	C10H9N
229	1,2-Diethoxybenzene	—	—	—	0.51	—	19.01	2050-46-6	C10H14O2
230	4H-1,2,4-Triazol-4-amine	—	—	—	0.44	—	15.90	584–13-4	C2H4N4
231	1H-Pyrrole-3,4-dicarbonitrile	—	—	—	0.42	—	17.70	125,666–25-3	C6H3N3
232	Propiolonitrile	—	—	—	—	2.97	8.15	1,070-71-9	C3HN
233	1-(Benzyloxy)-3,5-dinitrobenzene	—	—	—	—	1.28	10.52	128,923–97-7	C13H10N2O5
234	4-Methylesculetin	—	—	—	—	1.24	23.26	529–84-0	C10H8O4
235	4-Methyl-2,6-dihydroxyquinoline	—	—	—	—	0.99	16.52	34,982–01-9	C10H9NO2
236	1,3-bis(1,1-dimethylethyl)-benzene	—	—	—	—	0.96	16.52	1,014-60-4	C14H22
237	Acridine Orange	—	—	—	—	0.91	9.18	494–38-2	C17H19N3
238	2,2’-Bioxepane	—	—	—	—	0.88	19.65	74,793–02-5	C12H22O2
239	5-(2,2,2-Tri(2-cyanoethyl)acetyl)-2-methylpyridine	—	—	—	—	0.71	10.50	93,650–31-8	C17H18N4O
240	m-Aminophenylacetylene	—	—	—	—	0.68	17.70	54,060–30-9	C8H7N
241	7-Methyl-quinoline	—	—	—	—	0.67	19.84	612–60-2	C10H9N
242	1,2,5-Trimethylpyrrole	—	—	—	—	0.65	6.83	930–87-0	C7H11N
Total		8.54	13.57	21.85	17.42	17.40			

—, not detected.

### 2.8 Statistical analyses

All experiments were performed in triplicate. The results are presented as mean ± standard deviation. Data were analyzed using one-way analysis of variance (SPSS 26.0, IBM, USA), followed by Tukey’s post-hoc test. Differences were considered significant at *p* < 0.05. Response surface modeling was performed in Design-Expert 12 (Stat-Ease Inc., USA). Graphs were generated using OriginPro 2021 (Origin Laboratories Inc., Northampton, MA, USA).

## 3 Results

### 3.1 Changes in sensory attributes of Meitauza

**Figure 1:**
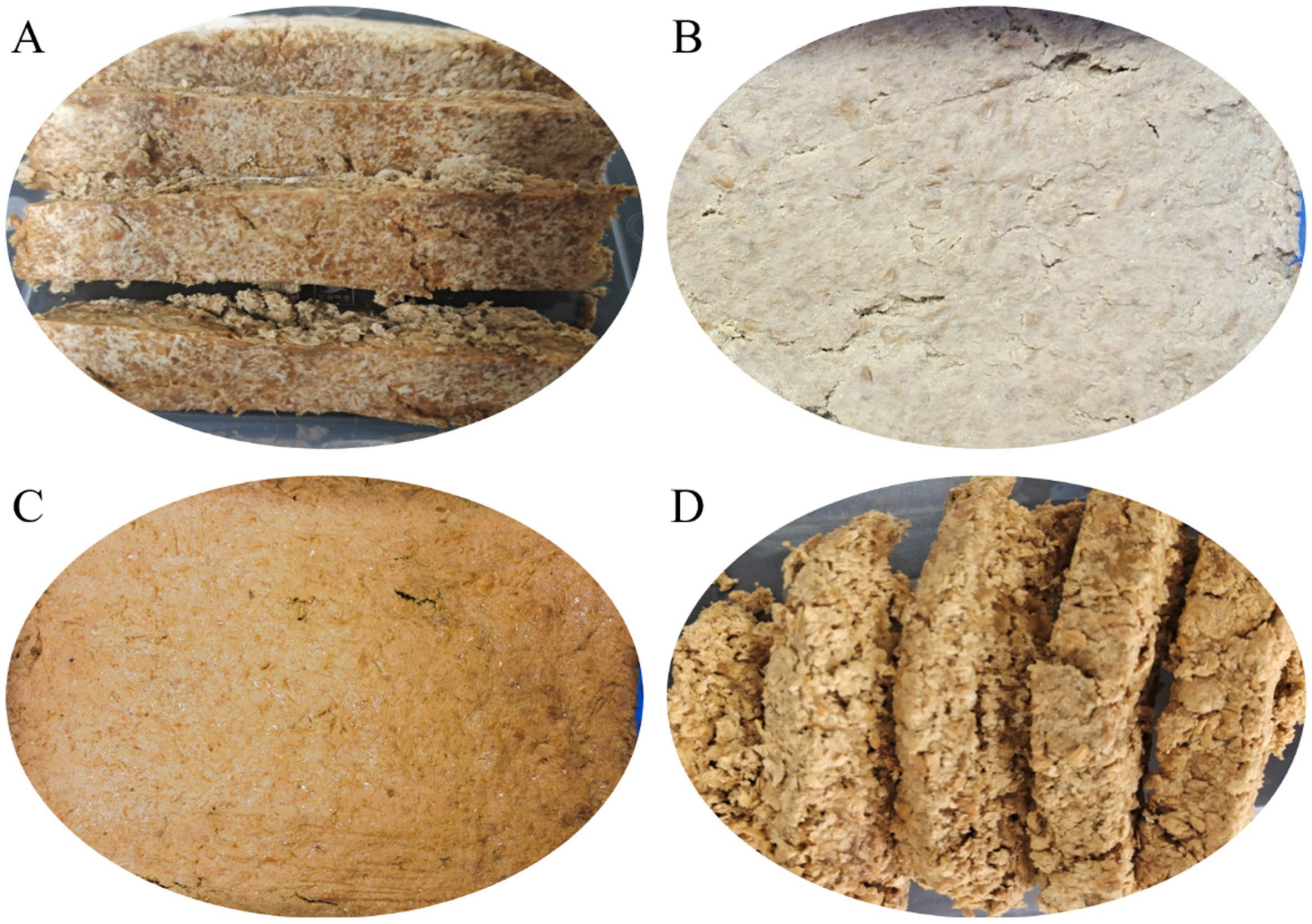
Visual appearance of fermented Meitauza samples. **(A)** *Mucor racemosus* Fresenius; **(B)** *Bacillus subtilis*; **(C)** *Mucor racemosus* Fresenius + *Bacillus subtilis*; **(D)** *Actinomucor elegans*.

The visual, aroma, and texture characteristics of Meitauza markedly varied depending on the inoculated strain. This indicates that microbial characteristics influence final product quality. Based on the sensory evaluation scores and SP levels, the optimal post-fermentation durations for MMtz, BMtz, MBMtz, and AMtz were established as 8, 4, 8, and 6 days, respectively. Quantitative overall acceptability scores (9-point hedonic scale, mean+/-SD, n = 30) at respective optimal time points: MBMtz Day8 = 7.2 ± 0.6(a), MMtz Day8 = 6.9 ± 0.5(a), TMtz = 6.3 ± 0.4(ab), BMtz Day4 = 5.8 ± 0.7(b), AMtz Day6 = 5.1 ± 0.8(c) (different letters: *p* < 0.05 Tukey HSD). Full data in Supplementary Table S10. Fixed-time (Day 8) comparison in Supplementary Table S11. The representative samples and sensory evaluation results are shown in Figure 1 and Supplementary Table S10. The MBMtz samples exhibited the most balanced sensory profile with a uniform surface color, compact texture, and rich aroma. In contrast, the AMtz samples exhibited a light color and a slightly alkaline odor.

### 3.2 Effects on SP and FAA

#### 3.2.1 MMtz samples

**Figure 2:**
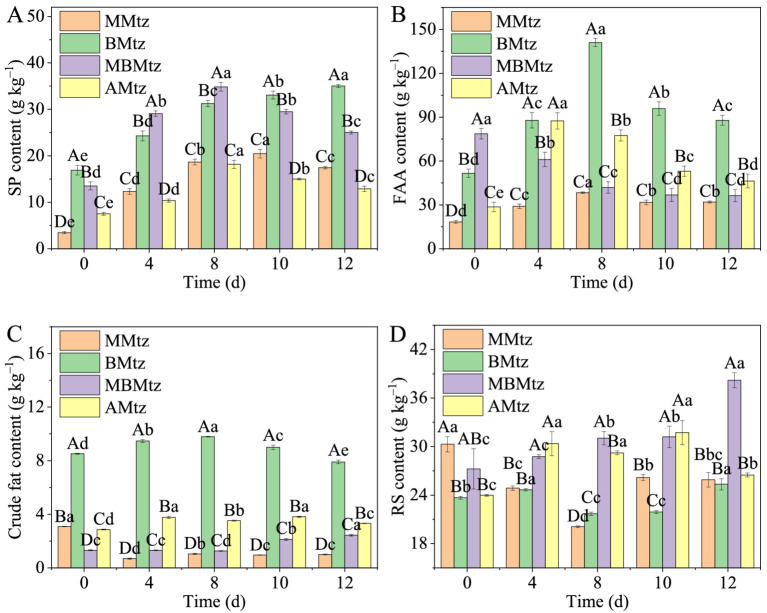
SP **(A)**, FAA **(B)**, crude fat **(C)**, and RS **(D)** contents in the soybean residue during fermentation.

During the pre-fermentation process, *M. racemosus* exhibited dynamic growth. After pre-fermentation, the soybean residue was shaped into blocks for the post-fermentation process at room temperature. The SP content was 3.467 g/kg at the end of pre-fermentation (Figures 2A,B). During the early post-fermentation period, the SP content gradually increased, reaching 18.67 g/kg on day 8 and peaking (20.46 g/kg) on day 10. The peak SP content in the post-fermentation period was three times higher than that in fresh soybean dregs. This can be attributed to *M. racemosus* protease, which hydrolyzes insoluble proteins, decreases their hydrophobicity, and increases their water solubility, enhancing the overall levels of SP in soybean residue.

#### 3.2.2 BMtz samples

During the pre-fermentation stage, the SP and FAA contents in the BMtz samples were 16.90 and 51.67 g/kg, respectively (Figures 2A,B), which were higher than those in fresh soybean dregs. This indicates that BMtz samples exhibited rich protein and amino acid contents. From days 0 to 8, the SP and FAA contents gradually increased to 31.25 and 141.00 g/kg, respectively. Two factors can explain the upregulation of SPs and FAAs. During the pre-fermentation stage, *B. subtilis* exhibits dynamic growth, accumulating amino acids and proteins. Additionally, the protease secreted by *B. subtilis* actively hydrolyzes insoluble proteins in soybean dregs, leading to the production of FAAs and SPs. However, the SP and FAA contents decreased after day 8. This can be attributed to the loss of moisture in soybean dregs, which inhibited the growth of *B. subtilis*, as well as to the depletion of available nutrients. Under these unfavorable conditions, *B. subtilis* can utilize existing proteins and amino acids to sustain metabolism, resulting in the depletion of SPs and FAAs.

#### 3.2.3 MBMtz samples

During the post-fermentation process, the FAA contents gradually declined from day 0 to day 8 but stabilized after day 8 (Figures 2A,B). This can be explained by the high microbial demand for amino acids during growth and reproduction. Protease activity hydrolyzed the low levels of insoluble proteins in soybean dregs, leading to a steady increase in SP content. These SPs were subsequently degraded into amino acids, partially offsetting microbial consumption and stabilizing amino acid levels. This may be the key mechanism driving the increase in SPs during the first 8 days and their subsequent decline.

On day 8, the SP and FAA contents in the MBMtz samples were 34.86 and 41.89 g/kg, respectively, which were significantly higher than those in fresh soybean dregs (4.59 and 27.44 g/kg, respectively). These results indicate that fermentation enhances protein solubilization and amino acid accumulation.

#### 3.2.4 AMtz samples

The contents of SPs and FAAs first increased and then decreased. However, the FAA content exhibited minor changes (Figures 2A,B).

The SP content was the highest on day 8 (18.16 g/kg). Meanwhile, the FAA content on day 8 was 77.44 g/kg. On day 4, the FAA content peaked (84.44 g/kg). However, the content of SPs was 10.36 g/kg on day 4. The protease activity of *A. elegans* can explain these findings. In particular, the protease activity releases amino acids during protein breakdown and hydrolyzes the proteins in soybean dregs. Some enzyme systems, such as transaminases, which facilitate the interconversion of amino acids, are produced during fermentation. Therefore, the release of amino acids through proteolysis and their synthesis or transformation through microbial metabolism increase FAA concentration. The total increase in FAAs during fermentation can be attributed to the collective functioning of these processes.

### 3.3 Effect on crude fat content

Post-fermentation changes in fat content are shown in Figure 2C. The fat content declined from day 0 to day 4 and subsequently stabilized in MMtz samples. Meanwhile, the fat contents on days 4 and 8 were 0.69 and 1.04 g/kg, respectively. This represented a 70% decrease from the initial fat content (3.09 g/kg). The decreased fat content can be attributed to the hydrolysis of fat into constituent fatty acids, which can improve the digestibility and nutritional quality of soybean residue.

The fat content in the BMtz samples initially increased and subsequently decreased. For example, the fat content increased from 8.52 g/kg on day 0 to 9.79 g/kg on day 8 and subsequently decreased to 7.91 g/kg on day 12. The fat contents in inoculated fermentation samples were significantly higher than those in fresh okara (2.5 g/kg) and approximately 5 times higher than those in traditional mold-fermented bean dregs (1.46 g/kg). This may be attributed to the enzymatic hydrolysis of nutrients by *B. subtilis* into low-molecular-weight compounds, such as alcohols and acids. Further esterification of these molecules results in the production of esters, which are isolated as crude fat with petroleum ether (Zeng et al., 2024).

During the early stages of fermentation, a slight reduction in fat content can be attributed to the activity of lipase, which hydrolyzes triglycerides into glycerol and fatty acids. Fatty acids are fat-soluble and can be extracted as crude fat. Thus, only glycerol is lost, leading to minimal overall changes. On day 12, the fat content increased from 1.32 g/kg (day 8) to 2.44 g/kg, indicating further metabolic activity of *B. subtilis*.

In AMtz samples, the fat content exhibited minimal variation (2.87–3.82 g/kg). This indicates that the fat content of soybean residue declines on the first 2 days of fermentation but is slightly altered thereafter. Stability can be attributed to decreased lipase production and activity due to the depletion of available fat (Wang J. et al., 2025).

### 3.4 Effect on RS content

RSs serve as essential substrates for microbial metabolism during fermentation and are involved in Maillard browning reactions that contribute to color and aroma development. The RS contents in different groups are shown in Figure 2D. In MMtz, the RS levels declined from 30.29 to 20.09 g/kg on day 8 but increased to 25.90 g/kg on day 12, indicating a dynamic balance between microbial utilization and polysaccharide hydrolysis. The RS levels in the BMtz samples remained constant (23.67–25.34 g/kg), suggesting equilibrium between sugar release and consumption by *B. subtilis*. In the MBMtz samples, the RS level continuously increased from 27.25 g/kg to 38.20 g/kg, which was 1.4-fold higher than the initial level and similar to that in TMtz. This can be attributed to the sequential activity of *M. racemosus* cellulases and *B. subtilis* amylases, generating abundant fermentable sugars. The RS content in the AMtz samples increased from 23.97 to 31.74 g/kg on day 8 but declined to 26.48 g/kg on day 12. This indicates initial hydrolysis and the subsequent sugar depletion for metabolic activity. Thus, the co-culture system produced the most favorable carbohydrate conversion pattern, supporting synergistic enzymatic activity between fungal and bacterial strains.

### 3.5 Effect on TA

**Figure 3:**
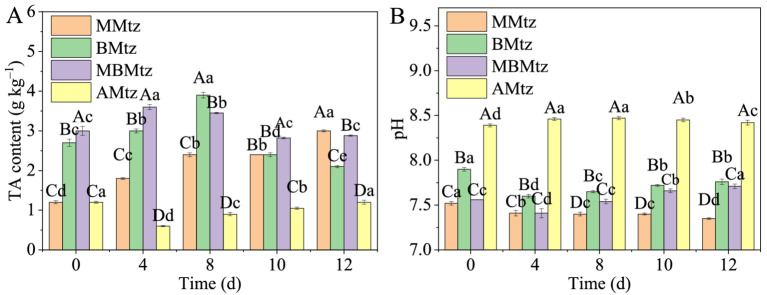
TA **(A)** content and pH **(B)** in the soybean residue during fermentation.

Post-fermentation changes in TA are shown in Figure 3A. In the MMtz samples, the TA increased from 1.20 g/kg on day 0 to 3.00 g/kg on day 12, which was consistent with organic acid accumulation in *M. racemosus*. Meanwhile, the TA of BMtz samples exhibited a transient peak (2.70 to 3.90 g/kg on day 4), followed by a decline (2.10 g/kg). This may be attributed to neutralization of acids by ammonia released from proteolysis. The TA of MBMtz samples exhibited moderate fluctuations (2.82–3.60 g/kg), indicating balanced acid–base metabolism between the two microbes. Meanwhile, the TA in AMtz samples initially decreased to 0.60 g/kg and then gradually recovered. This suggests early proteolytic alkalization, followed by mild acid production. These findings indicate that mixed inoculation moderates excessive acid or base accumulation, yielding chemical stability suitable for standardized product quality.

Figure 3B shows the post-fermentation pH changes in the four Meitauza samples. In MMtz samples, the post-pre-fermentation pH was 7.52, which gradually decreased to 7.45 on day 6 and to 7.35 on day 12. The pH in the BMtz samples progressively decreased from 7.90 on day 0 to 7.60 on day 4 but subsequently increased to 7.72 on day 10. This late increase in pH is due to the accumulation of alkaline end products, such as ammonia, released through protein hydrolysis. In MBMtz samples, the pH decreased slightly to 7.41 on day 4, which can be attributed to the microbial hydrolysis of carbohydrates and fats into fatty and organic acids.

Subsequently, the pH in MBMtz samples increased to 7.71 on day 12. The AMtz samples maintained an alkaline profile throughout fermentation with the pH in the range of 8.39–8.47. This is due to the virulent protease activity of *A. elegans*, which degrades the accessible protein in soybean residue, yielding alkaline end products, such as ammonia and amines (Ding et al., 2024). The accumulation of alkaline end products contributes to the high pH values of AMtz samples.

### 3.6 Effect on antioxidant capacity

**Figure 4:**
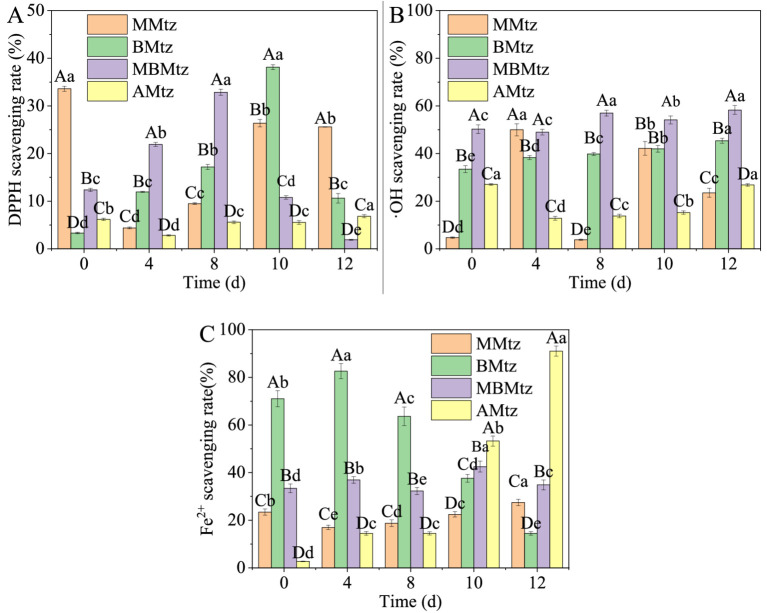
DPPH **(A)**, OH **(B)**, and Fe^2+^ **(C)** scavenging rate of four different Meitauza after fermentation.

The antioxidant activity of Meitauza samples was determined using the DPPH radical scavenging, hydroxyl radical scavenging, and ferric reducing antioxidant assays. The DPPH assay is based on the antioxidant-mediated reduction of the purple DPPH radical to light yellow hydrazine (Ali et al., 2025). The reduction power assay measures the conversion of ferricyanide (Fe^3+^) to ferrocyanide (Fe^2+^). Ferrocyanide further reacts with ferric chloride to form Prussian blue, which exhibits a maximum absorbance at 700 nm (Romero-Márquez et al., 2024). The absorbance is directly proportional to the reducing capacity of the sample. The antioxidant activity of Meitauza was evaluated using the DPPH and hydroxyl radical scavenging assays. Additionally, the iron-reducing capacity of Meitauza samples was examined. Thus, this study quantified the ability of different samples to neutralize harmful reactive oxygen species (Figure 4).

The MMtz samples retained antioxidant activities post-fermentation. On day 0, the DPPH and hydroxyl radical scavenging activities were 33.63 and 4.76%, respectively, while the iron-reducing power was 23.45%. Fermentation increased the hydroxyl radical scavenging rate, which peaked on day 4 (50.00%). However, the DPPH scavenging rate declined to 4.40%, while the iron-reducing power decreased to 17.00% on day 4. The iron-reducing activity exhibited the least fluctuation (17.00 and 27.45%). On day 12, the antioxidant activities of the samples stabilized (DPPH scavenging rate: 25.59%; hydroxyl scavenging rate: 23.53%; iron-reducing power: 27.45%).

The BMtz samples exhibited distinct antioxidant activities. The DPPH scavenging activity steadily increased, peaking (38.14%) on day 8. The hydroxyl radical scavenging activity peaked (42.01%) on day 8. However, the iron-reducing power peaked early (82.60% on day 2) but steadily declined to 37.64% on day 8.

The antioxidant activity of co-culture systems (MBMtz) was higher than that of monoculture systems. The DPPH and hydroxyl scavenging activities first increased and then decreased. On day 8, the DPPH and hydroxyl scavenging activities were 12.40 and 50.28%, respectively, while the iron-reducing power was 33.40%. Meanwhile, the DPPH scavenging, hydroxyl scavenging, and iron-reducing activities on day 8 were 32.87, 57.00, and 32.30%, respectively.

The AMtz samples exhibited weak antioxidant activities. The DPPH scavenging and hydroxyl radical scavenging activities were constant during post-fermentation, indicating minimal activity. However, the iron-reducing power increased significantly in the later stage, reaching 53.28 and 91.02% on days 8 and 12, respectively.

### 3.7 Effect on volatile flavor components

**Figure 5:**
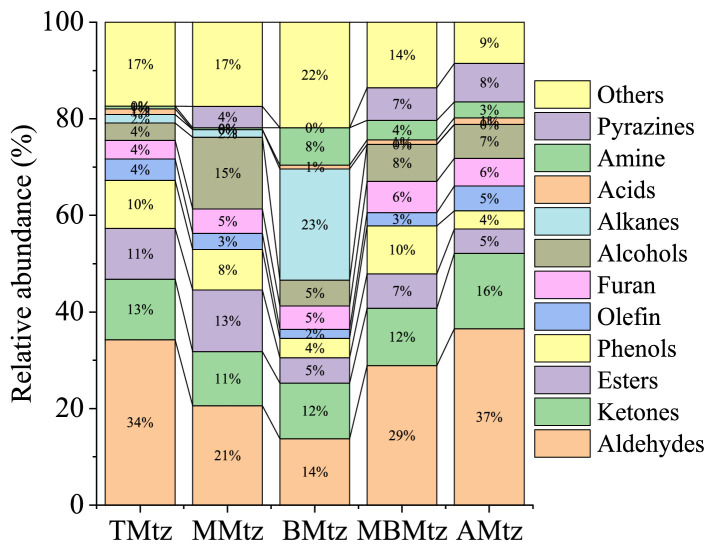
The composition and relative contents of volatile flavor compounds in Meitauza after fermentation.

The flavor components of BMtz, MMtz, MBMtz, and AMtz were compared with those of TMtz obtained from Hubei Province. The composition and contents of volatile flavor substances and the distribution of relative contents of different volatile components are presented in Table 2 and Figure 5. The types and relative proportions of volatile flavor compounds varied between the samples. This indicates that fermentation of soybean residue with different pure microbial strains results in distinct volatile profiles, leading to distinct changes in the flavor characteristics of Meitauza.

The following volatile flavor compounds (n = 242) were detected in the samples: 31 esters, 24 alcohols, 38 ketones, 6 phenols, 22 aldehydes, 6 acids, 5 furans, 4 pyrazines, 12 amines, 23 alkanes, 15 olefins, and 56 other types. Esters were detected in all five samples. The relative contents of esters in MMtz (7.1%), BMtz (5.27%), MBMtz (12.77%), and AMtz (10.53%) samples were higher than those in TMtz samples (5.02%). The MBMtz samples exhibited the highest relative contents of alcohols (14.86%). Meanwhile, the relative content of alcohols in TMtz was 7.01%. The relative content of alcohols was the lowest in the AMtz samples (3.59%). Ketones were the most frequently detected volatiles. The relative content of ketones in TMtz samples was 15.61%, while that in MMtz, AMtz, and MBMtz samples was 11.5%. The AMtz samples exhibited the highest relative content of ketones (12.56%). The relative contents of phenolic compounds in TMtz, MMtz, BMtz, MBMtz, and AMtz samples were 3.77, 9.95, 4.01, 8.39, and 9.91%, respectively. Compared with those in the TMtz samples, the phenolic compound contents were 2.6, 1.1, 2.2, and 2.6 times higher in MMtz, BMtz, MBMtz, and AMtz samples, respectively. The relative contents of aldehydes in MMtz, BMtz, MBMtz, and AMtz samples were 36.54, 28.91, 13.73, 20.59, and 34.24%, respectively. Meanwhile, the relative contents of acids in TMtz, BMtz, MBMtz, and AMtz samples were 1.15, 0.83, 0.91, and 1.35%, respectively. The MMtz samples did not comprise acids. The contents of furans in BMtz, MMtz, and MBMtz samples were 7.71, 4.08, and 3.32%, respectively, but were low in TMtz and AMtz samples. Pyrazines were detected in BMtz, MBMtz, and AMtz samples, with the BMtz samples exhibiting the highest total pyrazine content (23.00%): 2,5-dimethylpyrazine (14.42%), 2,3,5-trimethylpyrazine (6.56%), 3-ethyl-2,5-dimethylpyrazine (1.11%), and 2,6-diethylpyrazine (0.91%). This is consistent with *B. subtilis* Maillard reactions at 37 degrees C. AMtz contained only 1.76% total pyrazines; MBMtz contained 1.62%. [Correction: an earlier version incorrectly attributed 23% pyrazine content to AMtz.] A comprehensive numerical cross-check of all percentage values cited in the text against Table 2 was performed; two additional minor transcription errors were identified and corrected (highlighted in yellow in the revised manuscript). In this study, 12 amines were detected in the five samples. The relative content of amines was the highest in TMtz samples (5.16%), followed by AMtz samples (4.44%). The relative contents of amines in the other three groups were in the range of 1.86–3.34%. Volatile alkanes and olefins were also detected in.

## 4 Discussion

### 4.1 Conventional component index

The effect of different microbial strains on the post-fermentation quality of Meitauza was evaluated by examining sensory attributes, nutritional value, physicochemical parameters, and volatile flavor compounds (Ding et al., 2024). Fermentation significantly altered the sensory profile and nutrient balance, generating several volatile compounds that imparted a distinctive flavor to Meitauza.

The FAA (38.3 g/kg), SP (18.7 g/kg), and RS contents (20.1 g/kg) in MMtz samples were 3.8, 13.4, and 8 times higher than those in fresh soybean residue, respectively (Liu et al., 2024; Heng et al., 2022). The fat content in MMtz samples declined to 1.0%, representing a 70% reduction. The BMtz sample comprised the highest SP and FAA contents. This can be attributed to the high protease activity of *B. subtilis*. The contents of SP and FAAs in AMtz samples were higher than those in TMtz samples. This is because of the microbial hydrolysis of soybean proteins into peptides and low-molecular-weight proteins, which enhance solubility and protein availability. Extended fermentation allows for further breakdown of proteins into peptides and FAAs (Wen et al., 2022).

The apparent paradox of FAA values exceeding SP values (most notably BMtz: 141.00 vs. 31.25 g/kg) reflects a fundamental incompatibility between the two measurement systems. The Bradford assay detects proteins and polypeptides > = ~3–5 kDa and is blind to free amino acids and short peptides generated by *B. subtilis* proteases. The ninhydrin assay quantifies all primary alpha-amino groups (including individual amino acids and dipeptides) as leucine equivalents. These assays measure fundamentally different analyte pools; g/kg values are not directly comparable (Compton and Jones, 1985; Liu et al., 2023). The fat contents were high in BMtz samples, low in MMtz samples, and intermediate in MBMtz samples. This suggests that *B. subtilis* may comprise metabolic pathways for synthesizing or changing lipids.

Thus, inoculated fermentation provided products with increased levels of FAAs and SP when compared with the commercial TMtz benchmark. Additionally, inoculated fermentation enhanced the nutritional quality and palatability of soybean residue. [Pathogen risk claim removed: no microbiological safety data were collected. Future studies should include biogenic amine profiling, mycotoxin screening, and plate counts.].

### 4.2 Volatile flavor components

The flavor quality of Meitauza is closely associated with its volatile compounds. In particular, esters, alcohols, ketones, phenols, aldehydes, acids, furans, pyrazines, and amines shaped the aroma profile. The differential types and relative abundances of these volatiles imparted distinct sensory attributes to Meitauza, highlighting the influence of microbial inoculation on flavor formation. However, it must be noted that differences in volatile profiles between TMtz and the inoculated groups cannot be attributed solely to differences in microbial community composition. Fermentation temperature (15 °C for MMtz, 28 °C for AMtz, 37 °C for BMtz vs. ambient ~20–25 °C for TMtz), fermentation duration, moisture content, and substrate sterilization status all substantially influence volatile generation—particularly Maillard-derived compounds (pyrazines, furans), lipid oxidation products (aldehydes), and ester formation rates. In the absence of a controlled isotherm experiment or a matched laboratory spontaneous fermentation control, these confounding variables cannot be partitioned from microbial effects, and all inter-group flavor comparisons should be interpreted accordingly.

Esters were abundant in all samples and contributed to fruity and floral aromas. The esters detected in the samples included 2,2,4-trimethylpentanediol isobutyrate, *γ*-nonalactone, vinyl propionate, and vinyl n-hexanoate. The source of esters may be esterification reactions between lipid-derived alcohols and fatty acids, as well as carbohydrate fermentation pathways (Xiao Y. et al., 2023). The contents of esters in the inoculated fermentation groups were 1.4–2.5 times higher than those in the traditional fermentation group, enhancing the fruity aroma. Traditionally fermented Meitauza comprised seven esters, especially 4-[1-oxo-2-(1-pyrrolidyl) ethyl] aminobenzoate methyl ester (1.35%). In contrast, the inoculated fermentation samples comprised unique compounds, such as vinyl n-hexanoate (2.91% in Group A), γ-nonalactone (1.62% in AMtz samples and 2.04% in MMtz samples), and (Z)-propionate-3-hexyl ester (in MMtz samples). Isobutyl 2,2,4-trimethylpentanediol isobutyrate was abundant in all groups, except TMtz, indicating its potential role as a signature compound. The abundance of alcohols was low but contributed to the distinctive flavor profile (Romero-Márquez et al., 2024).

Traditionally fermented Meitauza comprised 1-nonene-3-ol, dodecyl alcohol, and (S)-(+)-5-methyl-1-heptanol with the relative contents of each exceeding 1% (Huang et al., 2022). In MMtz samples, the relative contents of four alcohols were higher than 1% with those of (E, Z)-3,6-nonadien-1-ol reaching 4%. Compounds, such as n-hexyl alcohol, 1-octen-3-ol, n-hexanal, and 2-amylfuran imparted a bean-like and mushroom-like aroma. The contents of 1-octen-3-ol and n-hexanal were high in MBMtz samples (10.93 and 3.8%, respectively), imparting fresh green aromas. Meanwhile, the contents of 1-nonene-4-ol were intermediate (0.96–1.44%) in MMtz, MBMtz, and AMtz samples. The BMtz samples comprised increased levels of 2-ethylcyclobutanol (1.67%) and 2-nonyl alcohol (1.3%). The AMtz samples comprised 1-nonene-3-ol (2.44%) and dodecyl alcohol (1.96%).

In this study, 16 ketones, which are associated with sweet and floral aromas, were detected. Traditional fermentation and inoculated fermentation groups exhibited differential ketone composition. The ketone types detected in different samples were as follows: MMtz samples: 3-octen-2-one and new derivatives, such as a diazo-ketone; BMtz samples: o-aminophenone and 2-butylcyclohexanone; MBMtz samples: 1-(2-furanyl)-2-butanone and 2-heptanone; AMtz samples: methyl nonyl ketone and 2-nonyl ketone. These ketones have a low odor threshold and can consequently influence Meitauza aroma even at trace levels (Chen et al., 2022; Wang P. et al., 2025).

Additionally, six phenols were detected in all samples. For example, 2,4-di-tert-butylphenol was consistently detected and is reported to exhibit broad-spectrum antimicrobial activity. However, 2,4-di-tert-butylphenol is a compound of regulatory concern in some food matrices. Its presence should not be interpreted as a safety benefit without dedicated toxicological assessment. Quantitative biogenic amine analysis, mycotoxin screening (aflatoxin B1/B2/G1/G2), and aerobic plate counts are needed before food safety conclusions can be drawn. Moreover, the contents of aldehydes, which were the most abundant volatiles, ranged from 13.7 to 36.5%. Aldehydes originate primarily from Strecker degradation and lipid oxidation and include nonylaldehyde, trans-2,4-decadienal, and trans-2-octenal (Xing and Yaylayan, 2024). Inoculated fermentation also generated compounds absent in traditionally fermented samples, such as benzaldehyde, trans-4-hexenal, and phenylacetaldehyde, which impart loquat-like and jasmine-like aromas, broadening the sensory profile (Wang P. et al., 2025).

The levels of acids were low in the inoculated fermentation groups. No acids were detected in MBMtz samples. The decreased levels of acids may contribute to an enhanced flavor profile. Furans, especially 2-N-amyl furan, were detected in all samples. The contents of furans were high in TMtz, MMtz, and BMtz samples, which conferred a sesame-like flavor. Pyrazines, which were highly abundant in BMtz samples (23.00% combined for 2,3,5-trimethylpyrazine, 2,5-dimethylpyrazine, 3-ethyl-2,5-dimethylpyrazine, and 2,6-diethylpyrazine), are important Maillard intermediates and confer roasted aroma and brown color (Xiao Q. et al., 2023; Deng et al., 2025). Additionally, the contents of amines in the inoculated fermentation group were lower than those in the traditional fermentation group. This may be due to reduced miscellaneous bacterial activity. This reduction minimized the risk of ammonia-like off-flavors; however, targeted biogenic amine quantification by HPLC is required before safety conclusions can be drawn. Hydrocarbons, which are primarily derived from lipid degradation, were detected, although their contribution to the sensory profile was limited (Fu et al., 2022). One unusual compound, 2,5-dimethylpyrimidine, was detected in BMtz samples (13.48%). However, the sensory role of 2,5-dimethylpyrimidine is unclear.

Inoculated fermentation consistently enriched esters (compounds with reported fruity character in similar fermented systems), characteristic ketones, and selective phenols and decreased undesirable acids, amines, and excessive phenolics Note: aroma descriptors inferred from GC–MS compound classes; GC-olfactometry or sensory panel validation required for confirmation (Zhao et al., 2024). These changes enhanced aroma complexity and improved stability. The key contributors to the distinct flavor included *γ*-nonalactone, 1-octen-3-ol, o-aminophenone, benzaldehyde, 2-N-amyl furan, and trimethylpyrazines. In summary, these findings indicate that microbial inoculation can be harnessed to customize the flavor quality of Meitauza, providing a scientific foundation for developing controlled fermentation strategies.

## 5 Conclusion

Inoculated fermentation markedly enhanced the nutritional composition, antioxidant capacity, and flavor diversity of Meitauza produced from soybean residue. Mixed fermentation with *M. racemosus* and *B. subtilis* yielded the best overall results, achieving the highest SP and RS levels and enhanced antioxidant performance, especially hydroxyl radical scavenging. This synergy between fungal and bacterial strains promoted efficient protein and carbohydrate hydrolysis and maintained balanced acidity. *B. subtilis* alone produced the highest increase in SP and FAA due to active protease secretion. However, the SP and FAA contents decreased during the late stages of fermentation due to nutrient depletion. *M. racemosus* facilitated acid production, while *A. elegans* maintained an alkaline environment, contributing to the production of distinct ammonia-based volatiles. GC–MS analysis identified 242 volatile compounds. Mixed fermentation was associated with the richest aroma profile characterized by high levels of esters and alcohols, imparting fruity and floral aromas. *B. subtilis* produced abundant pyrazines through Maillard reactions, potentially contributing to roasted and nutty aroma notes (pending validation by GC-olfactometry or trained sensory panel). These findings represent a controllable biotechnological process for sustainable and standardized Meitauza production at an industrial scale. Future studies should: (1) include biogenic amine profiling, mycotoxin screening, and pathogen counts for safety evaluation; (2) validate aroma descriptors by GC-olfactometry or trained panel; (3) use a laboratory-prepared spontaneous fermentation control instead of a commercial reference; (4) apply retention index verification and absolute quantification for all GC–MS volatiles.

## Data Availability

The original contributions presented in the study are included in the article/Supplementary material, further inquiries can be directed to the corresponding authors.

## References

[ref1] Ali E. M. Abdalameer N. K. Khalaph K. A. Hadi F. W. Ramadhan A. O. (2025). Green reduction approach for the synthesis of copper oxide nanoparticles using ginger extract and evaluation of their free radical scavenging activity. Eur. Phys. J. Plus 140:928. doi: 10.1140/epjp/s13360-025-06873-1

[ref2] Canaan J. M. M. Brasil G. S. A. P. de Barros N. R. Mussagy C. U. Guerra N. B. Herculano R. D. (2022). Soybean processing wastes and their potential in the generation of high value added products. Food Chem. 373:131476. doi: 10.1016/j.foodchem.2021.131476, 34731815

[ref3] Chen X. Lu Y. Zhao A. Wu Y. Zhang Y. Yang X. (2022). Quantitative analyses for several nutrients and volatile components during fermentation of soybean by *Bacillus subtilis* natto. Food Chem. 374:131725. doi: 10.1016/j.foodchem.2021.131725, 35021579

[ref9003] Compton S. J. Jones C. G. (1985). Mechanism of dye response and interference in the Bradford protein assay. Analytical Biochemistry, 151, 369–374. doi: 10.1016/0003-2697(85)90190-3 4096375

[ref4] Deng S. Cui H. Hussain S. Hayat K. Liu W. Zhang X. . (2025). Promoted formation of pyrazines by targeted precursor addition to improve aroma of thermally processed methionine-glucose Amadori compound. Food Chem. 465:142033. doi: 10.1016/j.foodchem.2024.142033, 39549517

[ref5] Ding Y. He W. Dai W. Xie X. Pan Y. Tang X. . (2024). Quality and flavor development of solid-state fermented surimi with Actinomucor elegans: a perspective on the impacts of carbon and nitrogen sources. Food Chem. 447:139053. doi: 10.1016/j.foodchem.2024.139053, 38518616

[ref6] Fu Y. Cao S. Yang L. Li Z. (2022). Flavor formation based on lipid in meat and meat products: a review. J. Food Biochem. 46:e14439. doi: 10.1111/jfbc.14439, 36183160

[ref7] Gao Y. Hu M. Meng W. Wen W. Zhang P. Fan B. . (2024). Study on the quality of soybean proteins fermented by *Bacillus subtilis* BSNK-5: insights into nutritional, functional, safety, and flavor properties. Food Chem. 443:138523. doi: 10.1016/j.foodchem.2024.138523, 38286093

[ref8] Heng X. Chen H. Lu C. Feng T. Li K. Gao E. (2022). Study on synergistic fermentation of bean dregs and soybean meal by multiple strains and proteases. LWT-Food Sci. Technol. 154:112626. doi: 10.1016/j.lwt.2021.112626

[ref9] Huang W. Deng Z. Lu L. Ouyang Y. Zhong S. Luo T. . (2022). Polysaccharides from soybean residue fermented by Neurospora crassa alleviate DSS-induced gut barrier damage and microbiota disturbance in mice. Food Funct. 13, 5739–5751. doi: 10.1039/D2FO00137C, 35527507

[ref10] Kong X. Su H. Shen H. Chen Y. Yang F. Zhang J. . (2025a). The combined use of 1-MCP and laser microporous film packaging maintains the quality of Shine Muscat grapes by inhibiting oxidative stress and cell wall catabolism. LWT-Food Sci. Technol. 225:117958. doi: 10.1016/j.lwt.2025.117958

[ref11] Kong X. Zhang J. Shen H. Shi N. Zhou H. Li Y. . (2025b). Screening, identification, and fermentation characteristics of lactic acid bacteria from pickled potherb mustard and potential applications. Foods 14:1431. doi: 10.3390/foods14081431, 40282832 PMC12026729

[ref9001] Lancioni C. Castells C. Candal R. Tascon M. (2022). Headspace solid-phase microextraction: Fundamentals and recent advances. Advances in Sample Preparation, 3, 100035. doi: 10.1016/j.sampre.2022.100035

[ref12] Li H. Li X. Liu Z. Chitrakar B. Liang Y. Hu L. . (2024). Fermentation of soybean residue by *A. auricula*: mechanisms and uses. J. Food Eng. 380:112138. doi: 10.1016/j.jfoodeng.2024.112138

[ref9002] Lim J. (2011). Hedonic scaling: A review of methods and theory. Food Quality and Preference, 22, 733–747. doi: 10.1016/j.foodqual.2011.05.008

[ref13] Liu D. Guo Y. Ma H. (2023). Production of value-added peptides from agro-industrial residues by solid-state fermentation with a new thermophilic protease-producing strain. Food Biosci. 53:102534. doi: 10.1016/j.fbio.2023.102534

[ref14] Liu L. Li J. Li S. Liu L. Wu B. Wang Y. . (2024). The potential use of *Zymomonas mobilis* for the food industry. Crit. Rev. Food Sci. 64, 4134–4154. doi: 10.1080/10408398.2022.2139221, 36345974

[ref15] Lu D. Zhang M. Wang F. Dai Z. Li Z. Ni J. . (2025). Nutritional value improvement of soybean meal through solid-state fermentation by proteases-enhanced Streptomyces sp. SCUT-3. Int. J. Biol. Macromol. 298:140035. doi: 10.1016/j.ijbiomac.2025.140035, 39828158

[ref16] Pang C. Xu W. Gan T. Gan T. Guo S. Chen Y. . (2023). Isolation, identification and enzyme producing capacity analysis of dominant strain in traditional fermented Meitauza. Sci. Tech. Food Ind. 44, 107–113. doi: 10.13386/j.issn1002-0306.2022050112

[ref17] Romero-Márquez J. M. Navarro-Hortal M. D. Forbes-Hernández T. Y. Varela-López A. Puentes J. G. Sánchez-González C. . (2024). Effect of olive leaf phytochemicals on the anti-acetylcholinesterase, anti-cyclooxygenase-2 and ferric reducing antioxidant capacity. Food Chem. 444:138516. doi: 10.1016/j.foodchem.2024.138516, 38306771

[ref18] Samarasiri M. Chen W. N. (2025). Solid-state fermentation with Pleurotus spp. on properties of soybean residue (Okara). Food Biosci. 74:107893. doi: 10.1016/j.fbio.2025.107893

[ref19] Shen H. Li Y. Song H. Bai J. Peng N. Ge X. . (2024). Quality improvement of soybean meal by simultaneous microbial fermentation and enzymolysis and untargeted metabolomic analysis of its metabolites. Food Biosci. 59:104090. doi: 10.1016/j.fbio.2024.104090

[ref20] Sui Y. Zhao X. Ding J. Sun S. Tong Y. Ma W. . (2025). A nondestructive and rapid method for in situ measurement of crude fat content in soybean grains. Food Chem. 491:144862. doi: 10.1016/j.foodchem.2025.144862, 40714491

[ref21] Tkaczewska J. Zając M. Jamróz E. Derbew H. (2022). Utilising waste from soybean processing as raw materials for the production of preparations with antioxidant properties, serving as natural food preservatives-a pilot study. LWT-Food Sci. Technol. 160:113282. doi: 10.1016/j.lwt.2022.113282

[ref22] Tuly J. A. Ma H. (2024). Bioconversion of food industrial waste okara by microbial fermentation: scope of omics study and possibility. Trends Food Sci. Tech. 146:104391. doi: 10.1016/j.tifs.2024.104391

[ref23] Usman M. Li Q. Luo D. Xing Y. Dong D. (2025). Valorization of soybean by-products for sustainable waste processing with health benefits. J. Sci. Food Agr. 105, 5150–5162. doi: 10.1002/jsfa.13999, 39498528

[ref26] Wang J. Yi G. Zhao T. Huang X. Zhao Y. Zhu M. . (2025). Jasmine tea: unveiling the secrets of processing, flavor characteristics, and potential health benefits. Crit. Rev. Food Sci. 66, 1752–1769. doi: 10.1080/10408398.2025.2556218, 40928510

[ref24] Wang P. Sui Q. Guo L. Deng X. Wang Z. Chen Y. . (2025). The evaluation of structural changes, in vitro digestibility and digestates composition of protein in soybean meal during high-temperature storage. J. Stored Prod. Res. 112:102640. doi: 10.1016/j.jspr.2025.102640

[ref25] Wang Y. Sun H. Han B. Li H. Y. Liu X. L. (2022). Improvement of nutritional value, molecular weight patterns (soluble peptides), free amino acid patterns, total phenolics and antioxidant activity of fermented extrusion pretreatment rapeseed meal with *Bacillus subtilis* YY-1 and *Saccharomyces cerevisiae* YY-2. LWT-Food Sci. Technol. 160:113280. doi: 10.1016/j.lwt.2022.113280

[ref27] Wen L. Bi H. Zhou X. Zhu H. Jiang Y. Ramadan N. S. . (2022). Structure and activity of bioactive peptides produced from soybean proteins by enzymatic hydrolysis. Food Chem. Adv. 1:100089. doi: 10.1016/j.focha.2022.100089

[ref28] Xiao Q. Huang Q. Ho C. T. (2023). Influence of deamidation on the formation of pyrazines and proline-specific compounds in Maillard reaction of asparagine and proline with glucose. J. Agric. Food Chem. 71, 7090–7098. doi: 10.1021/acs.jafc.3c00887, 37126799

[ref29] Xiao Y. Zhang S. Liu Z. Wang T. Cai S. Chu C. . (2023). Effect of inoculating *Pichia* spp. starters on flavor formation of fermented chili pepper: metabolomics and genomics approaches. Food Res. Int. 173:113397. doi: 10.1016/j.foodres.2023.113397, 37803735

[ref30] Xing H. Yaylayan V. (2024). Mechanochemistry of Strecker degradation: interaction of glyoxal with amino acids. Food Chem. 439:138071. doi: 10.1016/j.foodchem.2023.138071, 38061296

[ref31] Yu S. Huang Q. Hu W. Hui F. Ren Y. Chen X. . (2024). Potential prebiotic effects of soy by-products as novel dietary fibre: structure, function, in vitro simulation of digestion and fermentation properties. Int. J. Biol. Macromol. 278:134617. doi: 10.1016/j.ijbiomac.2024.134617, 39127293

[ref32] Zeng D. Cai Y. Liu T. Huang L. Wang J. Zhao M. . (2024). Effect of hydrophobic sucrose esters with different fat acid composition and esterification degree on whipped cream properties. Food Hydrocolloid. 146:109183. doi: 10.1016/j.foodhyd.2023.109183

[ref33] Zhang J. Chen R. Zhou H. Wen D. Lu Q. Xiong J. . (2024). Prevalence of aflatoxin B1 in four kinds of fermented soybean-related products used as traditional Chinese food. LWT-Food Sci. Technol. 191:115611. doi: 10.1016/j.lwt.2023.115611

[ref34] Zhao J. Lv Q. Liu M. Chen W. Li J. Qin Y. . (2024). Effect of co-inoculations of Saccharomyces cerevisiae and grape endophytes on the fermentation property, organic acid, and volatile aroma compounds. J. Food Sci. 89, 8296–8311. doi: 10.1111/1750-3841.17574, 39656747

