# Supplementary material for: Inoculation fermentation improves the nutritional quality and flavor profile of Chinese traditional fermented okara (Meitauza): a comparison with a commercial benchmark
Source: Front Microbiol. 2026 Jun 8;17:1846638. doi: 10.3389/fmicb.2026.1846638 (PMC13283960; doi:10.3389/fmicb.2026.1846638)
Supplement: Supplementary file 1 [file Table_1.DOC]

**Supplementary Materials 1**

**Table S1** Design factors and coded levels for response surface experiments of *Bacillus subtilis*

**Table S2** Design factors and coded levels for response surface experiments of *Mucor racemosus* f. racemosusf

**Table S3** Design factors and coded levels for response surface experiments of *Actinomucor elegans*

**Table S4** The results of protease activity analyses in Meitauza inoculated with *Bacillus subtilis*

**Table S5** Analysis of variance (ANOVA) for regression equations

**Table S6** The results of protease activity analyses

**Table S7** Analysis of variance (ANOVA) for regression equations

**Table S8** The results of protease activity analyses

**Table S9** Analysis of variance (ANOVA) for regression equations

**Table S10** Sensory evaluation results of Meitauza

**Table S1** Design factors and coded levels for response surface experiments of *Bacillus subtilis*

| Independent variables | Design  factors | Ranges and levels | | |
| --- | --- | --- | --- | --- |
|  |  | Low value (–1) | 0 | High value (+1) |
| Fermentation time (d) | A | 1 | 2 | 3 |
| Inoculation rate (%) | B | 1 | 3 | 5 |
| Water content (%) | C | 70 | 80 | 90 |

**Table S2** Design factors and coded levels for response surface experiments of *Mucor racemosus*

| Independent variables | Design  factors | Ranges and levels | | |
| --- | --- | --- | --- | --- |
|  |  | Low value (–1) | 0 | High value (+1) |
| Fermentation time (d) | A | 4 | 5 | 6 |
| Inoculation rate (%) | B | 1 | 2 | 3 |
| Water content (%) | C | 70 | 75 | 80 |

***Table S3*** *Design factors and coded levels for response surface experiments of Actinomucor elegans*

| Independent variables | Design  factors | Ranges and levels | | |
| --- | --- | --- | --- | --- |
|  |  | Low value (–1) | 0 | High value (+1) |
| Fermentation time (d) | A | 2 | 3 | 4 |
| Inoculation rate (%) | B | 2 | 3.5 | 5 |
| Water content (%) | C | 60 | 65 | 70 |

**Table S4** The results of protease activity analyses in Meitauza inoculated with *Bacillus subtilis*

| Run | Variables | | | Responses |
| --- | --- | --- | --- | --- |
|  | *A*  Fermentation time (d) | *B*  Inoculation rate (%) | *C*  Water content (%) | *Y*  Protease activity (U L^–1^) |
| 1 | 1 | 1 | 80 | 5.69 |
| 2 | 3 | 1 | 80 | 135.00 |
| 3 | 1 | 5 | 80 | 4.81 |
| 4 | 3 | 5 | 80 | 160.62 |
| 5 | 1 | 3 | 70 | 2.53 |
| 6 | 3 | 3 | 70 | 59.41 |
| 7 | 1 | 3 | 90 | 106.90 |
| 8 | 3 | 3 | 90 | 173.47 |
| 9 | 2 | 1 | 70 | 1.00 |
| 10 | 2 | 5 | 70 | 3.85 |
| 11 | 2 | 1 | 90 | 192.18 |
| 12 | 2 | 5 | 90 | 192.84 |
| 13 | 2 | 3 | 80 | 201.36 |
| 14 | 2 | 3 | 80 | 193.62 |
| 15 | 2 | 3 | 80 | 180.15 |

Note: Y=191.71+51.07A+3.53B+74.83C+6.63AB+2.42AC–0.5475BC–63.54A^2^–51.65B^2^–42.60C^2^

The optimal combination was 84.654 % initial water content of soybean residue, 1.876 % inoculation rate, and fermentation time 2.485 d.

**Table S5** Analysis of variance (ANOVA) for regression equations

| Source | Sum of Squares | DF | Mean Square | F Value | *P*-Value |
| --- | --- | --- | --- | --- | --- |
| Model | 93391.89 | 9 | 10376.88 | 7.60 | 0.0189* |
| A | 20866.18 | 1 | 20866.18 | 15.29 | 0.0113* |
| B | 99.76 | 1 | 99.76 | 0.07 | 0.7977 |
| C | 44790.24 | 1 | 44790.24 | 32.82 | 0.0023** |
| AB | 175.56 | 1 | 175.56 | 0.13 | 0.7345 |
| AC | 23.47 | 1 | 23.47 | 0.02 | 0.9008 |
| BC | 1.20 | 1 | 1.20 | 0.00 | 0.9775 |
| A^2^ | 14904.72 | 1 | 14904.72 | 10.92 | 0.0214* |
| B^2^ | 9848.15 | 1 | 9848.15 | 7.22 | 0.0435* |
| C^2^ | 6699.87 | 1 | 6699.87 | 4.91 | 0.0776 |
| Residual | 6823.87 | 5 | 1364.77 |  |  |
| Lack of Fit | 6593.47 | 3 | 2197.82 | 19.08 | 0.0502 |
| Pure Error | 230.40 | 2 | 115.20 |  |  |
| Cor Total | 100200.00 | 14 |  |  |  |
| *R*^2^=0.9319 | *R2 Adj*=0.8093 |  | | | |

Note:*, Significant (*p* <0.05); **, Significant (*p* <0.01); ***, Significant (*p* <0.001).

**Table S6** The results of protease activity analyses in Meitauza inoculated with *Mucor racemosus Fresenius*

| Run | Variables | | | Responses |
| --- | --- | --- | --- | --- |
|  | *A*  Fermentation time (d) | *B*  Inoculation rate (%) | *C*  Water content (%) | *Y*  Protease activity (U L^–1^) |
| 1 | 4 | 1 | 75 | 31.89 |
| 2 | 6 | 1 | 75 | 60.22 |
| 3 | 4 | 3 | 75 | 39.23 |
| 4 | 6 | 3 | 75 | 59.30 |
| 5 | 4 | 2 | 70 | 22.94 |
| 6 | 6 | 2 | 70 | 40.40 |
| 7 | 4 | 2 | 80 | 39.82 |
| 8 | 6 | 2 | 80 | 60.92 |
| 9 | 5 | 1 | 70 | 20.15 |
| 10 | 5 | 3 | 70 | 38.35 |
| 11 | 5 | 1 | 80 | 54.06 |
| 12 | 5 | 3 | 80 | 52.60 |
| 13 | 5 | 2 | 75 | 61.53 |
| 14 | 5 | 2 | 75 | 64.92 |
| 15 | 5 | 2 | 75 | 58.09 |

Note: Y=61.51+10.87A+2.89B+10.70C–2.06AB+0.9100AC–4.92BC–7.06A^2^–6.79B^2^–13.43C^2^

The optimal combination was 76.343 % initial water content of soybean residue, 2.278 % inoculation rate, and fermentation time 5.718 d.

**Table S7** Analysis of variance (ANOVA) for regression equations

| Source | Sum of Squares | DF | Mean Square | F Value | *P*-Value |
| --- | --- | --- | --- | --- | --- |
| Model | 2951.45 | 9 | 327.94 | 25.94 | 0.0011** |
| A | 945.26 | 1 | 945.26 | 74.77 | 0.0003*** |
| B | 67.05 | 1 | 67.05 | 5.30 | 0.0695 |
| C | 915.06 | 1 | 915.06 | 72.38 | 0.0004*** |
| AB | 17.06 | 1 | 17.06 | 1.35 | 0.2979 |
| AC | 3.31 | 1 | 3.31 | 0.26 | 0.6305 |
| BC | 96.63 | 1 | 96.63 | 7.64 | 0.0396* |
| A^2^ | 184.12 | 1 | 184.12 | 14.56 | 0.0124* |
| B^2^ | 170.31 | 1 | 170.31 | 13.47 | 0.0144* |
| C^2^ | 666.13 | 1 | 666.13 | 52.69 | 0.0008*** |
| Residual | 63.21 | 5 | 12.64 |  |  |
| Lack of Fit | 39.89 | 3 | 13.30 | 1.14 | 0.4987 |
| Pure Error | 23.32 | 2 | 11.66 |  |  |
| Cor Total | 3014.67 | 14 |  |  |  |
| *R*^2^=0.9790 | *R2 Adj*=0.9413 |  | | | |

Note:*, Significant (*p* <0.05); **, Significant (*p* <0.01); ***, Significant (*p* <0.001).

**Table S8** The results of protease activity analyses in Meitauza inoculated with *Actinomucor elegans*

| Run | Variables | | | Responses |
| --- | --- | --- | --- | --- |
|  | *A*  Fermentation time (d) | *B*  Inoculation rate (%) | *C*  Water content (%) | *Y*  Protease activity (U L^–1^) |
| 1 | 3 | 3.5 | 65 | 9.28 |
| 2 | 4 | 3.5 | 60 | 107.34 |
| 3 | 2 | 5 | 65 | 97.14 |
| 4 | 3 | 5 | 60 | 143.45 |
| 5 | 2 | 3.5 | 60 | 16.92 |
| 6 | 3 | 3.5 | 65 | 130.02 |
| 7 | 4 | 5 | 65 | 117.03 |
| 8 | 2 | 3.5 | 70 | 99.34 |
| 9 | 3 | 2 | 70 | 57.80 |
| 10 | 3 | 2 | 60 | 111.67 |
| 11 | 3 | 5 | 70 | 138.02 |
| 12 | 3 | 3.5 | 65 | 144.33 |
| 13 | 4 | 3.5 | 70 | 159.30 |
| 14 | 2 | 2 | 65 | 147.20 |
| 15 | 3 | 3.5 | 65 | 131.56 |
| 16 | 3 | 3.5 | 65 | 127.38 |
| 17 | 4 | 2 | 65 | 135.96 |

Note: Y=140.28+29.97A+23.02B+22.79C–12.94AB–32.70AC–11.89BC–36.55A^2^–14.43B^2^–12.90C^2^

The optimal combination was 67.712 % initial water content of soybean residue, 4.314 % inoculation rate, and fermentation time 3.071 d.

**Table S9** Analysis of variance (ANOVA) for regression equations

| Source | Sum of Squares | DF | Mean Square | F Value | *P*-Value |
| --- | --- | --- | --- | --- | --- |
| Model | 28894.22 | 9 | 3210.47 | 13.10 | 0.0013** |
| A | 7186.81 | 1 | 7186.81 | 29.33 | 0.0010** |
| B | 4238.90 | 1 | 4238.90 | 17.30 | 0.0042** |
| C | 4154.62 | 1 | 4154.62 | 16.96 | 0.0045** |
| AB | 669.52 | 1 | 669.52 | 2.73 | 0.1423 |
| AC | 4276.51 | 1 | 4276.51 | 17.45 | 0.0041** |
| BC | 565.49 | 1 | 565.49 | 2.31 | 0.1725 |
| A^2^ | 5625.62 | 1 | 5625.62 | 22.96 | 0.0020** |
| B^2^ | 876.13 | 1 | 876.13 | 3.58 | 0.1005 |
| C^2^ | 700.67 | 1 | 700.67 | 2.86 | 0.1347 |
| Residual | 1715.03 | 7 | 245.00 |  |  |
| Lack of Fit | 1044.27 | 3 | 348.09 | 2.08 | 0.2461 |
| Pure Error | 670.76 | 4 | 167.69 |  |  |
| Cor Total | 30609.24 | 16 |  |  |  |
| *R*^2^=0.9440 | *R2 Adj*=0.8719 |  | | | |

Note:*, Significant (*p* <0.05); **, Significant (*p* <0.01); ***, Significant (*p* <0.001).

**Table S10** Sensory evaluation results of Meitauza

| **Samples** | **Appearance** | **Odor** | **Texture** |
| --- | --- | --- | --- |
| MMtz | Uneven color inside and outside, with gray white visible on the surface and mostly brown inside; visible gray mycelium; Surface roughness and dryness. | Rich moldy aroma, and no peculiar smell. | The texture is relatively soft, the internal bonding is tight, the finger pressure is elastic, and it can be sliced. |
| BMtz | Uneven color inside and outside, with a light brown surface and a light yellow interior; no visible bacterial cells; Surface is moist and smooth. | There is a special odor caused by the fermentation of Bacillus subtilis, accompanied by a smell of decay. | Soft texture, no elasticity under pressure, difficult to slice and shape. |
| MBMtz | Uniform color inside and outside, brown in color; visible gray mycelium; surface is relatively rough. | Moldy aroma mixed with a special odor. | Soft texture bed, difficult to slice and shape with no elasticity under finger pressure. |
| AMtz | The internal and external colors are uniformly slightly yellow; visible off white mycelium; surface is relatively dry. | The moldy aroma is strong, but mixed with ammonia odor. | The texture is relatively soft, elastic, and tightly bonded internally, which can be sliced. |

**Supplementary Materials 2**

**Figure S1** The effect of fermentation time on the protease (A), cellulase (B) activity, and free amino acid content of three inoculated soybean residues.

**Figure S2** The effect of inoculation rate on the protease (A), cellulase (B) activity, and free amino acid content of three inoculated soybean residues.

**Figure S3** The effect of water content on the protease (A), cellulase (B) activity, and free amino acid content of three inoculated soybean residues.

**Figure A1.** The effect of fermentation time on the protease (A), cellulase (B) activity, and free amino acid content of three inoculated soybean residues.

**Figure A2.** The effect of inoculation rate on the protease (A), cellulase (B) activity, and free amino acid content of three inoculated soybean residues.

**Figure A3.** The effect of water content on the protease (A), cellulase (B) activity, and free amino acid content of three inoculated soybean residues.
